# Angiogenesis-on-a-chip coupled with single-cell RNA sequencing reveals spatially differential activations of autophagy along angiogenic sprouts

**DOI:** 10.1038/s41467-023-44427-0

**Published:** 2024-01-03

**Authors:** Somin Lee, Hyunkyung Kim, Bum Suk Kim, Sehyun Chae, Sangmin Jung, Jung Seub Lee, James Yu, Kyungmin Son, Minhwan Chung, Jong Kyoung Kim, Daehee Hwang, Sung Hee Baek, Noo Li Jeon

**Affiliations:** 1 Interdisciplinary Program for Bioengineering, Seoul National University, Seoul, South Korea; 2 Institute of Advanced Machines and Design, Seoul National University, Seoul, South Korea; 3 Department of Biochemistry and Molecular Biology, Korea University College of Medicine, Seoul, South Korea; 4 BK21 Graduate Program, Department of Biomedical Sciences, Korea University College of Medicine, Seoul, South Korea; 5 Department of New Biology, DGIST, Daegu, South Korea; 6 Neurovascular Unit Research Group, Korea Brain Research Institute, Daegu, South Korea; 7 Department of Mechanical Engineering, Seoul National University, Seoul, South Korea; 8 Department of Life Sciences, Pohang University of Science and Technology (POSTECH), Pohang, South Korea; 9 School of Biological Sciences, Seoul National University, Seoul, South Korea; 10 Creative Research Initiatives Center for Epigenetic Code and Diseases, School of Biological Sciences, Seoul National University, Seoul, South Korea; 11 Qureator, Inc., San Diego, CA USA; 12 Present Address: Department of Radiation Oncology, Massachusetts General Hospital and Harvard Medical School, Boston, MA, USA

**Keywords:** Autophagy, Tissue engineering, Cellular signalling networks, Microfluidics, RNA sequencing

## Abstract

Several functions of autophagy associated with proliferation, differentiation, and migration of endothelial cells have been reported. Due to lack of models recapitulating angiogenic sprouting, functional heterogeneity of autophagy in endothelial cells along angiogenic sprouts remains elusive. Here, we apply an angiogenesis-on-a-chip to reconstruct 3D sprouts with clear endpoints. We perform single-cell RNA sequencing of sprouting endothelial cells from our chip to reveal high activation of autophagy in two endothelial cell populations- proliferating endothelial cells in sprout basements and stalk-like endothelial cells near sprout endpoints- and further the reciprocal expression pattern of autophagy-related genes between stalk- and tip-like endothelial cells near sprout endpoints, implying an association of autophagy with tip-stalk cell specification. Our results suggest a model describing spatially differential roles of autophagy: quality control of proliferating endothelial cells in sprout basements for sprout elongation and tip-stalk cell specification near sprout endpoints, which may change strategies for developing autophagy-based anti-angiogenic therapeutics.

## Abstract (web summary)

The functional heterogeneity of autophagy in endothelial cells during angiogenesis remains incompletely understood. Here, the authors apply a 3D angiogenesis-on-a-chip coupled with single-cell RNA sequencing to find distinct autophagy functions in two different endothelial cell populations during angiogenic sprouting.

## Introduction

Autophagy is a highly conserved and essential process for the degradation and recycling of intracellular organelles and materials^1–3^. How autophagy is involved in the maintenance of homeostasis and development of diseases has long been a fundamental question in biology and medicine. Recent studies have demonstrated the implication of autophagy in angiogenesis, the formation of new vascular capillaries during organ development or under pathological conditions. For instance, the increased autophagy in endothelial cells (ECs) under heat-denaturation, hypoxia, or glucose-starved conditions leads to angiogenic phenotypes such as enhanced cell migration and proliferation^4–6^. On the other hand, various anti-cancer drugs resulted in an anti-angiogenic effect accompanied by activation of autophagy^7–9^. Several different functions of autophagy in sprouting ECs have been reported, which are associated with proliferation, differentiation, and migration of ECs^7^.

Such different functions can be attributed to functional heterogeneity of autophagy in sprouting ECs. The ECs in the angiogenic environment have been categorized into tip, stalk, and phalanx cells. Phalanx cells are quiescent, but undergo differentiation to stalk cells upon angiogenic stimuli. Stalk and tip cells are characterized not only by the relative positions along elongating sprouts, but also by the migratory or proliferative phenotype, which is associated with distinct metabolic status and activation of different signaling pathways^8–11^. It has been considered that the distinct metabolic status may cause differential autophagic potential between stalk and tip cells^8^. Previous studies have also demonstrated that stalk cells are present in the basement and also near the endpoints of elongating sprouts, and these two groups may also exhibit heterogeneous autophagic activations. However, the single-cell level investigation has been rarely performed to decipher cellular heterogeneity of the autophagic potential along angiogenic sprouts, which may propose a model linking the autophagic heterogeneity to differential functions of tip and stalk cells.

To precisely investigate cellular heterogeneity of autophagy in sprouting ECs, the angiogenesis model should be able to distinguish ECs with distinct autophagic potential at the single-cell level during angiogenic sprouting. However, in vitro EC models widely used in the previous autophagy studies are limited to wound healing and tube formation assays that measure only migration or network formation ability, respectively^4–6,12–15^. Moreover, in vitro bead sprouting assays have been used, but generate only small numbers of sprouts growing in non-uniform directions. Meanwhile, in vivo vascular models may fully recapitulate cellular heterogeneity of autophagy. However, both the bead sprouting assays and in vivo vascular models have the limited experimental reproducibility and throughput in generation of angiogenic sprouts^6,12,15–17^, which may not be optimal for monitoring the single-cell level heterogeneity of autophagy during angiogenic sprouting. Recent advances in microfluidic organ-on-a-chips have enabled in vitro modeling of human 3D microvasculature under the gradient of pro-angiogenic factors^18–23^. Several models recapitulate the 3D structure of elongating sprouts (basement, middle, and leading positions of sprouts) toward the gradient source in highly reproducible and throughput manners, which are suitable to systematically explore single-cell level heterogeneity of autophagy in ECs along elongating sprouts.

Here, we applied an 3D angiogenesis-on-a-chip model recently developed by our group^24,25^ that can recapitulate human 3D angiogenic sprouting through co-culture of human umbilical vein endothelial cells (HUVECs) and fibroblasts, the latter of which release pro-angiogenic factors to activate the HUVECs for angiogenic sprouting. To investigate the heterogeneity of autophagy in ECs along elongating sprouts, we then performed single-cell RNA sequencing (scRNA-seq) analysis of ECs collected from the sprouts on the chip. Clustering and trajectory analyses of scRNA-seq data identified distinctive populations of ECs with high activation of autophagy in the basement and leading positions of the sprouts. Finally, we selected genes defining the spatially differential activations of autophagy along elongating sprouts and then examined the effects of the differential autophagic activation on angiogenic sprouting by performing sprouting experiments with HUVECs after knockdown of these genes using our advanced chip model.

## Results

### Microfluidic angiogenesis-on-a-chip reveals high activation of autophagy near endpoints of angiogenic sprouts

**Fig. 1:**
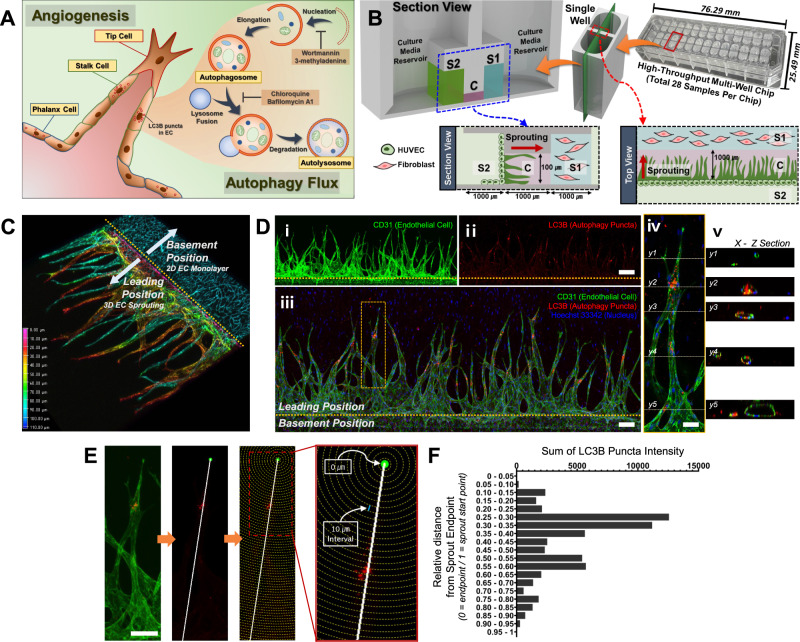
Microfluidic angiogenesis-on-a-chip showing spatial activation of autophagy along elongating sprouts. **A** Schematic illustration describing ECs (tip, stalk, and phalanx cells) in elongating sprouts during angiogenesis and autophagy processes in ECs. **B** Schematic overview of chip design for 3D angiogenesis in vitro assay. Channels S1, S2, and C contain fibroblasts secreting VEGF, ECs, and elongating sprouts, respectively. **C** Reconstructed 3D confocal image of angiogenic sprouting at day 4 after EC loading. 3D depth coated image indicates each 3D sprout positioned in a different depth point. Color bar, the gradient of depth. **D** Representative z-projected 3D confocal image of angiogenesis-on-a-chip at day 4 with immunostaining of CD31 (green, i), LC3B (red, ii), and Nucleus (blue, iii). A sprout in yellow-dotted box (iii) is depicted in a higher magnification (iv), and the orthogonal view of the sprout represents its 3D lumen structure (v). Scale bars, 200 (ii), 100 (iii), and 50 μm (iv). Four times of experiments was repeated independently with similar results. **E** Schematic illustration of image processing for estimating the relative distance of LC3B autophagy puncta from the endpoint of the sprout. Line connecting the sprout endpoint and LC3B punctum and 10 μm-interval contours centered at the endpoint are shown. Scale bar, 100 μm. **F** Sum of the intensities of LC3B puncta located at each indicated interval of the relative distance of LC3B puncta from the endpoints of 23 sprouts. Source data are provided as a Source Data file.

To effectively evaluate the activation of autophagy in ECs along elongating angiogenic sprouts at the single-cell level (Fig. 1A), we utilized a high-throughput 3D multi-cellular culture chip with the spontaneous capillary flow-driven patterning of cellular hydrogel to reproducibly recapitulate angiogenic sprouting^24–26^. Each chip consists of 28 wells each of which is designed to have the size of two wells in a commercial 384-well plate and compartmentalized into three channels (C, S1, and S2) without a physical barrier such as a micro-post structure (Fig. 1B). Channel C and S1 are initially patterned with 3D acellular fibrin hydrogel and fibroblast-embedded fibrin hydrogel, respectively, while Channel S2 is patterned with a monolayer of HUVECs in the side contiguous to Channel C. Based on the chemotactic gradient of pro-angiogenic factors secreted from fibroblasts in Channel S1, the loaded HUVECs in Channel S2 initiate angiogenic sprouting uniformly in the horizontal direction into Channel C toward the gradient in an interstitial flow setting (1.99 μm/sec with hydrostatic difference = 80 μL at day 1 and 1.4 μm/sec with hydrostatic difference = 40 μL at day 2) to promote angiogenic sprouting (Supplementary Fig. 1). Sufficient numbers of sprouts for statistical evaluation of autophagic activation in the sprouts are generated in three to four days after EC loading (Fig. 1C), and the distributions of both sprout length and width were found to be consistent across different samples under the same condition (Supplementary Fig. 2), indicating both throughput and reproducibility of our chip model in angiogenic sprouting.

Immunofluorescence analysis using the antibody of LC3B, an autophagy marker for autophagy activation^27^, revealed that the ECs highly expressing LC3B were located near sprout endpoints (Fig. 1D). To further quantitatively analyze the distribution pattern of LC3B puncta along elongating sprouts, we developed a feature detection method that automatically detects sprout endpoints and LC3B puncta in individual sprouts from the confocal images (Fig. 1E). For each sprout containing LC3B puncta, this method draws a reference line passing through the sprout endpoint and the center-of-gravity of LC3B puncta, generates contours centered at the endpoint in the 10 µm-interval, and measures total intensity of pixels with LC3B immunofluorescence signals between two consecutive contours as abundances of LC3B puncta at the average distance of the two contours from the endpoint. For 23 sprouts in a sample, this method identified the distribution pattern of LC3B puncta along the distance from the sprout endpoint (Fig. 1F). The maximum intensity sum of LC3B puncta was shown in the defined 10 µm-intervals positioned between 25% and 30% distance from sprout endpoints. Taken together, these data demonstrate that autophagy is predominantly activated in ECs near the sprout endpoints, suggesting differential activation of autophagy in ECs along elongating sprouts.

### Autophagy inhibition leads to severe defects in angiogenic sprouting

**Fig. 2:**
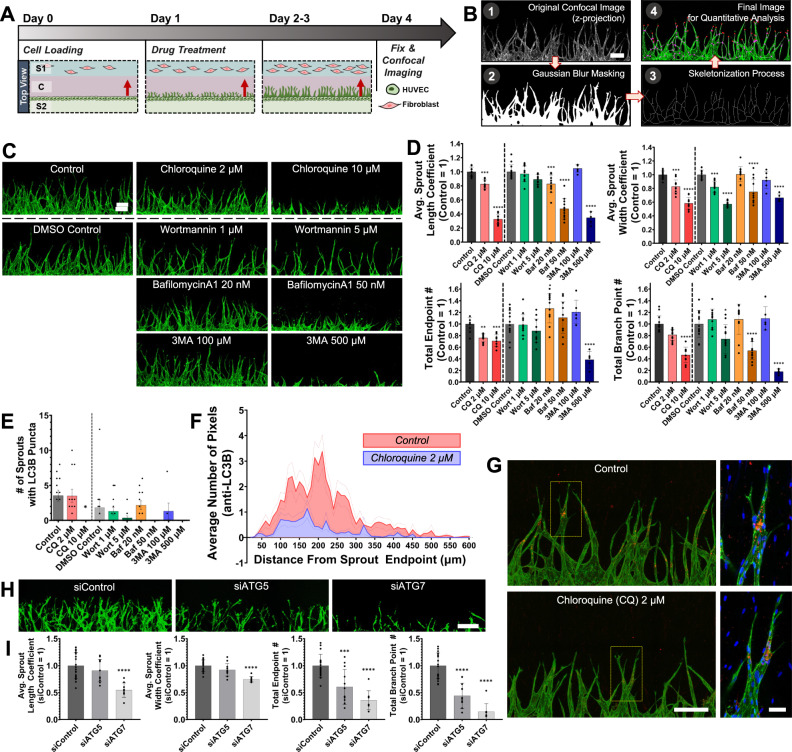
Reduced angiogenic sprouting by autophagy inhibition. **A** Temporal procedure of EC loading (day 0), autophagy inhibitor treatment (day 1), and fixation and imaging (day 4) during 3D angiogenic sprouting assay. **B** Image processing scheme for morphological feature detection from angiogenic sprouts. Scale bar, 200 μm. **C** Representative z-projected confocal image of sprouts immunostained with anti-CD31 (green) with or without treatment of autophagy inhibitors. Scale bar, 200 μm. Two times of experiments was repeated independently with similar results. **D** Quantitative analysis of the average sprout lengths, widths, endpoint counts, and branch point counts in the indicated inhibitor-treated conditions. *p < 0.05, **p < 0.01, ***p < 0.001, ****p < 0.0001, One-way ANOVA with Tukey’s posthoc tests. *n* = 13 per condition with Control, DMSO Control, and Wort 1 μM; *n* = 12 per condition with CQ 2 μM, Wort 5 μM, Baf 20 nM, and Baf 50 nM; *n* = 10, 6, 5 for CQ 10 μM, 3MA 100 μM, 3MA 500 μM, respectively. Biological replicates. Data are mean ± S.D. Source data and exact *p*-values are provided in a Source Data file. **E** Quantification of the numbers of sprouts with LC3B puncta in the indicated conditions. *p < 0.05, One-way ANOVA with Tukey’s posthoc tests. *n* value is same as the data shown in Fig. 2D. Biological replicates. Data are mean ± S.D. Source data and exact *p*-values are provided in a Source Data file. **F** Distribution of the average number of pixels with intensities of LC3 puncta over their distances from sprout endpoints in samples treated with (131 sprouts from 5 biological replicate samples) or without (181 sprouts from 7 biological replicate samples) 2 μM CQ. Data are mean ± S.E.M. Source data are provided as a Source Data file. **G** Representative z-projected confocal images of angiogenic sprouts with or without CQ treatment. Immunostained CD31 (green), LC3B (red), and Nucleus (blue) are shown. A sprout in the yellow-dotted box (left; scale bar, 200 μm) is represented (right; scale bar, 50 μm) in higher magnification. **H** Representative z-projected confocal image of angiogenic sprouts with treatment of siControl, siATG5, or siATG7. All samples are fixed in day 4 and stained with lectin (green). Scale bar, 200 μm. **I** Quantitative analysis of the average sprout lengths, widths, endpoint counts, and branch point counts in the ECs transfected with siControl (*n* = 24), siATG5 (*n* = 12), or siATG7 (*n* = 10). Biological replicates. ***p < 0.001, ****p < 0.0001, One-way ANOVA with Tukey’s posthoc tests. Data are mean ± S.D. Source data and exact *p*-values are provided in a Source Data file.

Our microfluidic angiogenesis-on-a-chip system has also the capability to add the modulators of angiogenic sprouting to evaluate the effects of the materials on angiogenic sprouting. The predominant activation of autophagy near sprout endpoints suggests potential regulatory effects of autophagy on angiogenic sprouting. To examine this, we analyzed the effects of the following four autophagy inhibitors on the morphology of sprouts: wortmannin (Wort) and 3-methyladenine (3MA) that inhibit the nucleation of autophagosome, and chloroquine (CQ) or bafilomycin A1 (Baf) that inhibit the fusion of autophagosome and lysosome. A single treatment of each inhibitor was performed through both Channels S1 and S2 for the inhibitor to effectively reach all ECs along elongating sprouts at the following concentrations adopted from the previous studies using the 2D culture of ECs (Supplementary Table 1)^27–35^ CQ (2 or 10 μM), Wort (1 or 5 μM), Baf (20 or 50 nM), and 3MA (100 or 500 μM). Compared to the 2D culture systems, however, our chip generates larger numbers of angiogenic sprouts (Supplementary Fig. 2). Due to this difference, we determined the treatment time as day 1 after EC loading, the time when we started to observe sufficient numbers of emerging sprouts (Fig. 2A, day 1) and LC3 puncta to ensure statistical power in evaluation of the inhibitor effects, and then obtained the confocal images at day 4 after EC loading to evaluate the changes in the morphology of sprouts (Fig. 2A, day 4).

To quantitatively analyze the morphological changes in angiogenic sprouting by the autophagy inhibitors, we developed another feature detection method that performs Z-projected image generation, Gaussian blur masking, skeletonization process, and extraction of the morphological features (Fig. 2B) to determine distributions of length, width, endpoint count, and branch point count for the sprouts observed in the confocal images. The distributions of the morphological features showed that CQ, Baf, and 3MA decreased significantly (P < 1.0 × 10^−3^) the length, width, and branch point count of sprouts in dose-dependent manners while Wort tended to decrease those parameters, but such changes in the length and branch point count were not statistically significant (Fig. 2C, D). For the sprout endpoint count, CQ and 3MA led to the significant dose-dependent decreases, and Wort tended to decrease it with no statistical significance. In contrast, Baf rather increased the average sprout endpoint. Nevertheless, these data indicated that the inhibitors led to severe reductions in angiogenic sprouting. We next quantitatively analyzed the distribution of LC3B puncta after treatment of the autophagy inhibitors, and the numbers of LC3B puncta-containing sprouts tended to reduce in dose-dependent manners (Fig. 2E). We then analyzed the distribution of the endpoint-to-LC3B puncta distance for the samples treated with 2 μM CQ and found that CQ significantly decreased the frequency of LC3B puncta over the entire range of the endpoint-to-LC3B puncta distance (Fig. 2F, G). These reductions were consistent with those in the morphological features of the sprouts. Of note, the reduced LC3B by CQ appears to be counter-intuitive compared to the previous findings. One possible explanation is that autophagy inhibition might prune populations of ECs that express LC3B and are dependent on autophagy, which can be supported by the previous finding that CQ has possibly harmful effects on viability of ECs^36^.

The pharmacological inhibition of autophagy may result in the reduced sprouting indirectly through fibroblasts. To address this issue, we further conducted genetic inhibition of autophagy by siRNA-mediated knockdown of ATG5 and ATG7, two core autophagy-related genes during the canonical autophagy process, in EC^37,38^. Knockdown of ATG7 reduced sprout length, width, endpoint, and branch point, and knockdown of ATG5 reduced endpoint and branch point (Fig. 2H, I). These results were consistent with those observed above after treatment of autophagy inhibitors (Fig. 2C, D). These knockdown data indicate that the reduced sprouting is likely not due to indirect effect of autophagy inhibitors on fibroblast. Taken together, these results from both chemical and genetic inhibition of autophagy collectively suggest potential roles of autophagy in promoting angiogenic sprouting occurring in our 3D angiogenesis-on-a-chip.

### scRNA-seq identifies tip- and stalk-like ECs and proliferating ECs

**Fig. 3:**
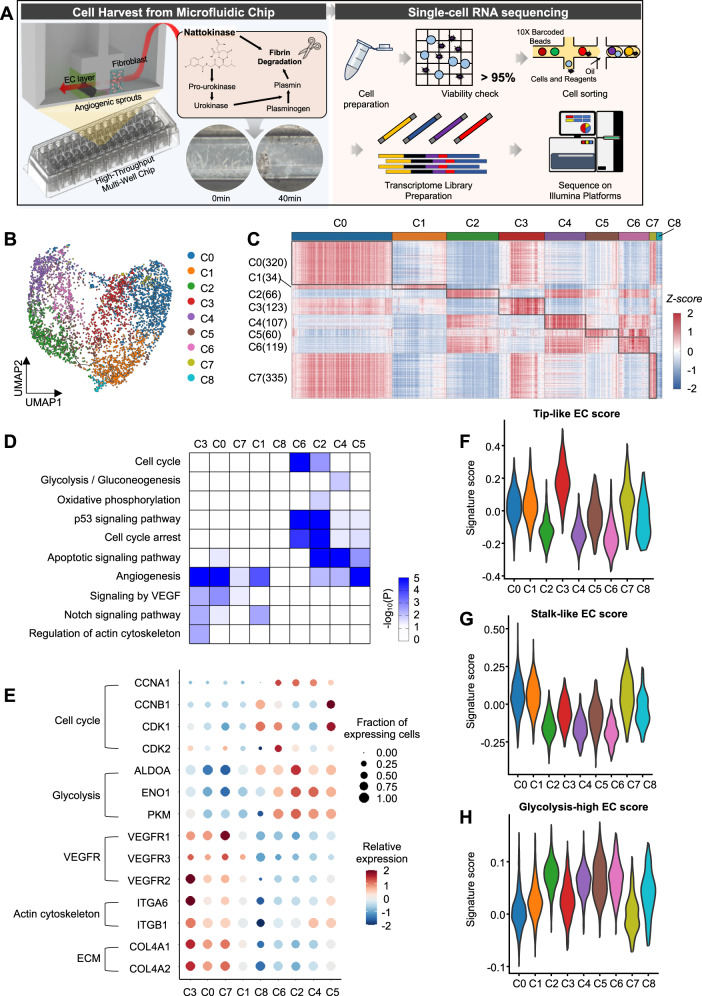
Cellular heterogeneity of human ECs in angiogenic sprouts. **A** Schematic overview of scRNA-seq analysis of ECs involving collection of 3D angiogenic sprouts in the microfluidic chips, viability check, library construction, and sequencing. **B** UMAP plot of ECs with and without Fibroblast (FB) condition. Cells are colored by their clusters (see the color legend in the right). **C** Heat map showing marker genes of clusters. The number of marker genes is presented in parenthesis. **D** Cellular pathways enriched by marker genes of each cluster. Enrichment significance (enrichment *P*-value from hypergeometric test implemented at ConsensusPathDB) for cellular pathways is displayed as –log10(P). Color bar, the gradient of –log10(P). **E** Dot plot showing the relative expression of marker genes associated with cell cycle, glycolysis, VEGF receptors, actin cytoskeleton, and ECM pathways. Violin plot showing the distribution of the tip-like (**F**) and stalk-like (**G**) cell scores and glycolysis signature scores (**H**). Source data are provided as a Source Data file.

The predominant activation of autophagy near sprout endpoints indicates the heterogeneity of autophagy in ECs along the elongating sprouts in our chip. To systematically investigate the heterogeneity of autophagy, we next performed scRNA-seq of ECs collected from the 3D angiogenesis-on-a-chip using nattokinase on day 5 after EC loading in the presence of fibroblasts. The scRNA-seq analysis was also performed for ECs in the same experimental setting, but without fibroblasts as a control (Fig. 3A). We profiled a total of 22,077 cells from the two conditions (9891 and 12,186 cells with and without fibroblasts, respectively) based on our quality control criteria (Supplementary Fig. 3A, B). Two distinct cell populations representing ECs and fibroblasts are observed (Supplementary Fig. 3C, D). We focused on the EC population (2372 and 12,186 ECs with and without fibroblasts) in the subsequent analyses to examine the heterogeneity of autophagy in ECs along elongating sprouts.

After correcting cell-cycle effects, we performed clustering analysis of ECs separately in each condition and identified 6 clusters (FP0-5) and 9 clusters (FA0-6) of ECs in the presence and absence of fibroblasts, respectively, using Louvain clustering (Supplementary Fig. 3E, F). To compare clusters of tip- and stalk-like ECs between the two conditions, we evaluated signature scores for these clusters using genes previously reported to be highly elevated in tip-like (38 genes) and stalk-like (26 genes) ECs^39^. FP4 and FA4 exhibited higher tip-like scores than the other clusters in the two conditions, respectively, while FP2-3, and FA0, 2, and FA9 showed higher stalk-like scores (Supplementary Fig. 3G, H), indicating that the same marker genes appear to define tip- and stalk-like ECs in the two conditions. However, the proportions of tip-and stalk-like ECs were different between FP and FA conditions [e.g., tip-like FP4 (16.5%) and FA4 (12.8%)] (Supplementary Fig. 3I). Nonetheless, considering the shared signature scores of tip- and stalk-like ECs between the two conditions, we decided to combine the datasets generated from the two conditions to enhance statistical power in the subsequent clustering and trajectory analyses. To avoid biases toward a higher number of ECs in the absence of fibroblasts, we sampled a subset of these ECs with a comparable size to ECs captured in the presence of fibroblasts when combining the ECs from the two conditions (Supplementary Fig. 3J).

After correcting batch effects between two EC populations, clustering analysis of the combined ECs identified 9 clusters (C0-8) (Fig. 3B). To examine functional characteristics of C0-8, we identified marker genes predominantly upregulated in each cluster (Fig. 3C) and then cellular pathways represented by the marker genes using enrichment analysis of cellular pathways and gene ontologies with ConsensusPathDB^40^ (Fig. 3D). C3 was strongly associated with VEGF/NOTCH signaling pathways (Fig. 3D and VEGFR1-3, ITGA1/6, and COL4A1-2 in Fig. 3E) and migration-related actin cytoskeleton and extracellular matrix (ECM) organization pathways. Correspondingly, when the signature scores of tip- and stalk-like ECs were evaluated, C3 exhibited higher scores of tip-like ECs (Fig. 3F). C0 and C1 were associated with angiogenesis and VEGF/NOTCH signaling pathways (Fig. 3D, E), and showed higher signature scores of stalk-like ECs (Fig. 3G), together with C7 and C8. These data suggest that C3 and C0-1 can be considered tip- and stalk-like ECs, respectively. Comparison of the combined and individual clustering results showed that tip-like clusters in the presence (FP4) and absence (FA4) of fibroblasts were clustered into the same cluster C3 (FP4 vs. FA4 for C3, Jaccard similarity = 0.68), and stalk-like clusters in the two conditions (FP2-3 and FA0, 2, and 9) were mainly into C0 (FP2 and FA0 and 2; FP2 vs. FA0/2 for C0, Jaccard similarity = 0.77) or C1 (FP3 and FA2 and 9; FP3 vs. FA2/9 for C1, Jaccard similarity = 0.66) (Supplementary Fig. 3K).

Finally, C2 and C4-6 were associated with the pathways related to cell proliferation (cell cycle and apoptosis in Fig. 3D). Interestingly, the representative genes in glycolysis (ALDOA, ENO1, and PKM in Fig. 3E) were highly elevated in these clusters, which was diluted in Fig. 3D due to their shared expression patterns between these clusters. Moreover, when the scores were estimated using glycolysis-related genes (200 genes in MSigDB), these clusters showed higher glycolysis scores than the other clusters (Fig. 3H). These data suggest that C2 and C4-6 appear to be activated proliferating ECs with high levels of glycolysis. Comparison of the combined and individual clustering results showed that more non-tip- or stalk-like EC clusters were identified in the absence of fibroblasts (FA1, 3, and 5-8), which were mainly included in C2 and C4-6 (Supplementary Fig. 3K), than in the presence of fibroblasts (FP0-1 and 5). Taken together, the unbiased inclusion of these shared tip/stalk-like clusters and distinctive non-tip/stalk-like clusters in the combined clusters suggest that there is likely no significant masking of the differences between the two conditions in the combined clustering.

### Autophagy shows spatially differential activation patterns in the basement and leading positions of sprouts

**Fig. 4:**
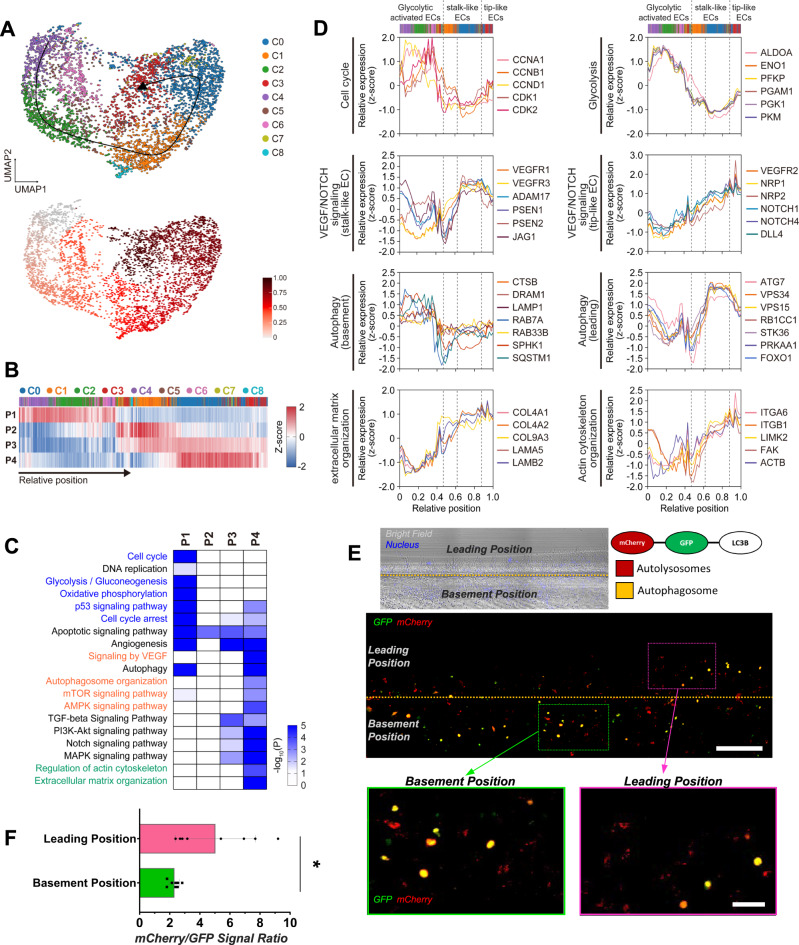
Spatially differential activations of autophagy in the basement and leading positions of elongating sprouts. **A** UMAP plots showing the trajectory (up) and corresponding relative positions (down) along elongating sprouts. Linear trajectory is obtained from ECs in C0-8. Cells are colored by their clusters (see the color legend in the right). **B** Four sets of genes (P1-4) showing activation in basement (P1), middle (P2), and leading positions (P3-4) along the trajectory. The mean expression (Z-score) of genes in each set is displayed in individual ECs along the trajectory. Colored bars on the top denote cluster memberships of the individual ECs. Relative position along the trajectory increases along the elongating sprouts axis (arrow). **C** Cellular pathways enriched by the genes in P1-4 for the trajectory. Enrichment significance (enrichment *P*-value from hypergeometric test implemented at ConsensusPathDB) for cellular pathways is displayed as –log10(P). Color bar, the gradient of –log10(P). **D** Relative expression profiles of the indicated representative genes (see the gene legend in the right) involved in the indicated pathways related to cell proliferation, angiogenesis, autophagy, and tip cell migration along the trajectory. **E** Representative z-projected images of 3D angiogenic sprouts generated from ECs transfected with LC3B-mCherry-GFP at day 2. The overall distribution of LC3B puncta within a sample is shown in above (Scale bar, 200 μm). The subregions in the leading (magenta box) and basement (green box) positions of elongating sprouts are magnified in below (Scale bar, 50 μm). Red and yellow, mCherry+ and mCherry+GFP + LC3B puncta, respectively. **F** The ratio (right) of mCherry+ to GFP + LC3B signal in the basement and leading positions of elongating sprouts in eight samples (Biological replicates). *p < 0.05, unpaired two-sided t-test. Data are mean ± S.D. Source data and exact *P*-values are provided in a Source Data file.

Cells in C0-8 are distributed along elongating sprouts (basement, middle, or leading positions) and are differentially associated with tip-stalk selection, tip cell migration, and stalk cell proliferation/sprout elongation. To infer relative distributions of the cells in C0-8 along elongating sprouts, we next performed trajectory analysis and found a linear trajectory that started at proliferating ECs (C4) and ended at tip-like ECs (C3; Fig. 4A). Since the above analyses suggested no significant masking of important differences between the two conditions in the combined clustering, we carried out the trajectory analysis using ECs used for the combined clustering. To examine functional roles of C0-8 along elongating sprouts, we then identified four groups of genes showing activation in the basement (P1), middle (P2), and leading (P3-4) positions of the elongating sprouts (Fig. 4B; Supplementary Data 1A). The enrichment analysis of cellular pathways and gene ontologies revealed that cell proliferation and glycolysis were activated in ECs (P1) at the basement (Fig. 4C, blue labeled), suggesting their association with stalk cell proliferation/sprout elongation. Interestingly, angiogenesis and autophagy were activated in ECs at both the basement (P1) and leading (P3 or 4) positions. The core pathways of angiogenesis (VEGF/NOTCH signaling) and autophagy (autophagosome organization and mTOR/AMPK signaling) were predominantly activated in the leading position (P4; Fig. 4C, orange labeled), consistent with the predominant expression of LC3B puncta near sprout endpoints. Moreover, ECM and actin cytoskeleton organization pathways, known to be associated with tip cell migration, were also predominantly activated in the leading position (P4; Fig. 4C, green labeled). All these data suggest the association of ECs with high autophagy potential near sprout endpoints with tip-stalk selection occurring in the leading position. Finally, we checked whether these findings included potential artifacts caused by combining ECs in the presence and absence of fibroblasts. To this end, we performed the above analyses (Supplementary Fig. 4A–D) using only ECs in the presence of fibroblasts. These analyses revealed that the following major findings were still valid only with ECs in the presence of fibroblasts (Supplementary Fig. 4C, D), indicating no apparent artifactual conclusions for the major findings from the combined analysis of ECs in the two conditions: 1) dual activation of autophagy at the basement (FP_P1 in Supplementary Fig. 4C) and leading position (FP_P3); 2) increased cell proliferation and glycolysis at the basement (FP_P1); and 3) predominant activation of angiogenesis (FP_P3 and 4) and ECM/actin cytoskeleton (FP_P4) in the leading position. Interestingly, however, due to the higher number of tip-like ECs in the presence of fibroblasts (Supplementary Fig. 3K), FP_P4 showing tip-like cell-specific expression was identified (Supplementary Fig. 4B), which was not apparent in P1-4 (Fig. 4B). Although many genes were shared, P1-4 and FP_P1-4 included still different sets of genes (Supplementary Data 1B), which explains different gene lists of genes in Fig. 4D and Supplementary Fig. 4D.

To investigate potential functional associations of autophagy with the above pathways, we then compared expression patterns of key genes in the pathways along the trajectory (i.e., elongating sprouts). The tip-like ECs (C3) had high expression levels of 1) genes related to migration and cytoskeleton dynamics (Figs. 4D), 2) VEGFR2 and NRP1/2 [Fig. 4D, VEGF/NOTCH signaling (tip-like ECs)], and 3) previously reported tip cell markers (Supplementary Fig. 5A), compared to those in stalk-like (C0-1) and proliferating ECs (C2 and C4-6), supporting the validity of the tip-like ECs. Also, the proliferating ECs showed high expression levels of cell cycle genes, supporting the involvement of proliferating ECs in sprout elongation (Fig. 4D and Supplementary Fig. 5A). Stalk-like cells, particularly in C0, showed higher expression levels of 1) VEGFR1, 2) JAG1 that inhibits NOTCH signaling in tip cells, and 3) ADAM17 and γ-secretases (PSEN1/2) that activate NOTCH signaling in stalk cells [Fig. 4D, VEGF/NOTCH signaling (stalk-like ECs)], supporting the validity of the stalk-like ECs. We next identified autophagy-related genes from P1 and 3 by which autophagy pathway was enriched (Fig. 4C), according to KEGG (autophagy pathway), WikiPathways (autophagy pathway), Reactome (autophagy and macroautophagy), and gene ontology biological processes (Autophagy and autophagosome organization). Intriguingly, two groups of them showed spatially differential activations: 1) genes highly activated in proliferating ECs in the basement sprouts (Fig. 4D, Autophagy-basement; Supplementary Fig. 5B); and 2) genes that showed the increased expression in the stalk-like ECs, compared to in proliferating ECs, but the decreased expression in the tip-like ECs (Fig. 4D, Autophagy-leading; Supplementary Fig. 5C).

The autophagic activation in the leading position was confirmed in Figs. 1 and 2. To verify the distribution of LC3B puncta in sprout basements, we thus transfected ECs with the mCherry-GFP-tagged LC3B construct where the GFP tag is acid-sensitive while the mCherry tag is acid-insensitive, and fusion of autophagosomes to late endosomes or lysosomes thus results in red fluorescence while autophagosomes show yellow fluorescence. Sprouting experiments using the transfected ECs in the 3D angiogenesis-on-a-chip confirmed the presence of mCherry+GFP+ (yellow) and mCherry+ (red) LC3B puncta in both the basement and leading positions of elongating sprouts (Fig. 4E), indicating the activation of autophagy in the two regions. Interestingly, more LC3B puncta was observed in the basement than in the leading positions (Fig. 4E), but the ratio of mCherry+ to GFP + LC3B signal was significantly higher in the leading position than in the basement (Fig. 4F). These data suggest that the autophagosome formation more frequently occurs in the basement while the autophagic flux to autolysosome is stronger in the leading position. Of note, the notable presence of LC3B puncta in Fig. 4E could appear to be inconsistent with those shown in Fig. 1D (iii) and 2G due to the smaller depths of the basement in these previous images. To address this inconsistency issue, we have newly performed 3D angiogenic sprouting experiments followed by LC3B immunostaining and found that new images, which correspond to Fig. 1D (iii), with larger depths of the basement confirmed the presence of notable amounts of LC3B puncta in the basement (Supplementary Fig. 5D, E).

To examine whether our findings are still valid in vivo, we next re-analyzed a previously reported scRNA-seq dataset generated from tumor tissues of patients with breast cancer^41^. After clustering ECs in this dataset (Supplementary Fig. 6A), we categorized them into tip-like EC (angiogenic and angiogenic LS), stalk-like EC (activated PCV and vein ii), and proliferating EC (capillary i, capillary venous, vein iii, and vein iv) clusters. We then separately performed the trajectory analysis for angiogenic LS and angiogenic clusters given the same proliferating and stalk-like EC clusters (Supplementary Fig. 6B). The trajectory analysis using angiogenic LS cluster revealed that the signature score patterns of the marker genes for the key pathways in Fig. 4D were consistent with those identified from our 3D system (Supplementary Fig. 6C, D), but inconsistent to those from the trajectory analysis using angiogenic cluster. The original study conjectured that angiogenic LS and angiogenic clusters might be early tip-like ECs transiting to tip cells and the mature tip cells, respectively. These results suggest that our 3D system is more prone to generate early tip-like ECs than the mature tip cells.

### Knockdown of autophagy regulators in stalk-like cells causes severe reduction in the number of tip cells in vitro

**Fig. 5:**
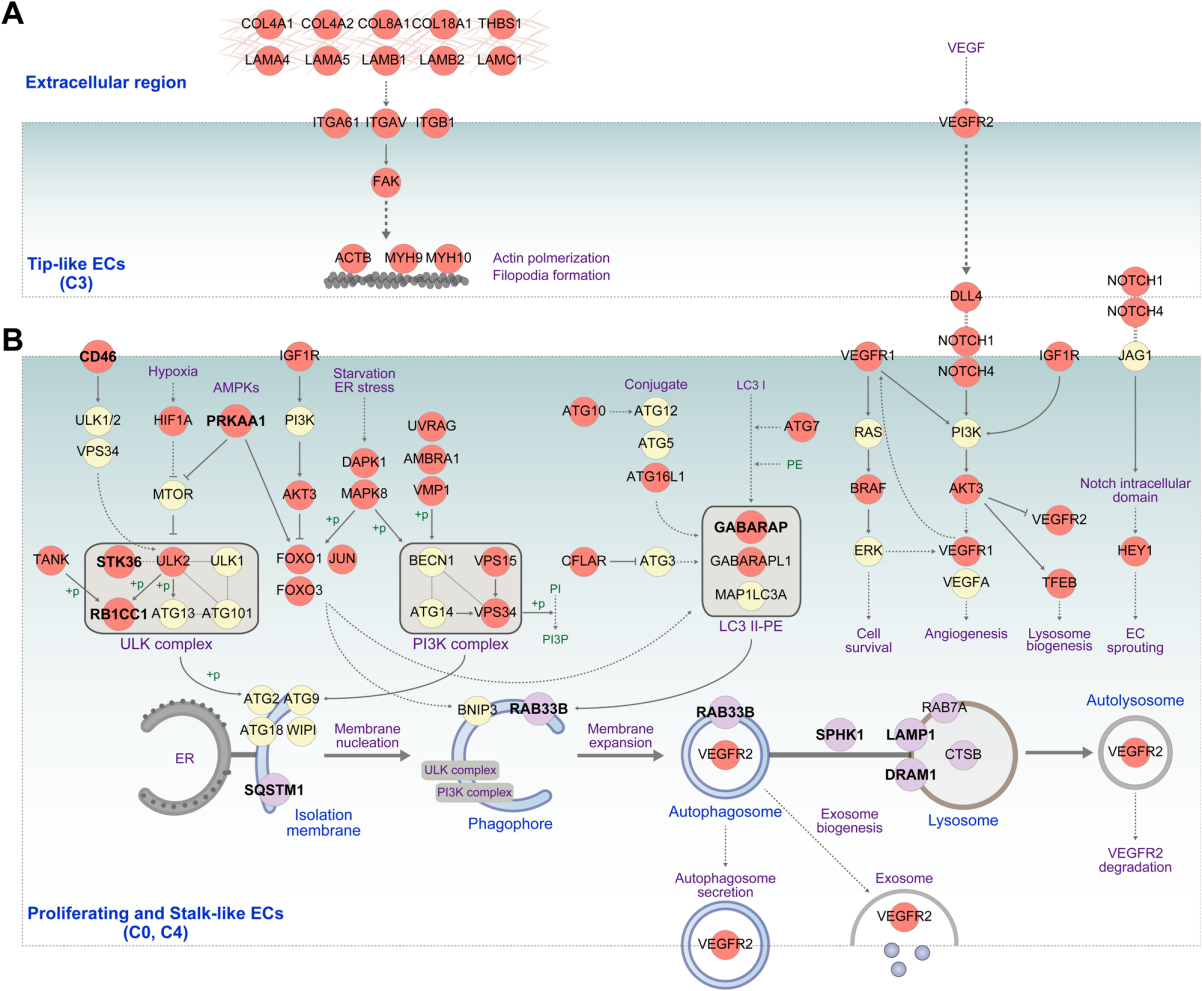
Network models describing interactions of spatially activated autophagy with tip-stalk specification and cell proliferation. Network models of the genes expressed in (**A**) tip-like ECs and (**B**) stalk-like and proliferating ECs. Node colors represent upregulation in the basement (magenta) and leading position (red) or no change (yellow) of the corresponding genes, respectively. Arrows and suppression symbols represent activation and inhibition in the corresponding signaling reactions, respectively. Solid and dotted lines denote direct and indirect interactions, respectively.

Autophagy has protective effects on EC survival in response to oxidative stress^42,43^ or under high glucose conditions^44^ through quality control to remove harmful substances. If autophagy was activated for quality control, however, there is no need for the aforementioned reciprocal activation pattern of autophagy between the stalk- and tip-like ECs in the leading position of the sprouts. Previously, autophagy was reported to play roles in VEGFR2 degradation^45^ and to increase the biogenesis of VEGFR2-carrying exosomes^46^. These data suggest that autophagy in the basement may be activated for quality control of proliferating ECs during sprout elongation while autophagy in the leading position may be activated for tip-stalk cell specification through regulation of VEGFR2 protein levels. To summarize these potential roles of autophagy, we constructed a network model describing regulatory relationships among 1) VEGF signalling in tip-like ECs (Figs. 5A), 2) VEGF/NOTCH signalling and the predicted regulation of VEGFR2 proteins by autophagy in stalk-like ECs (Figs. 5B), and 3) quality control in proliferating ECs by autophagy (Fig. 5B and Supplementary Fig. 7). The network model in Fig. 5B suggests a hypothesis that autophagy can decrease the protein level of VEGFR2 in the stalk-like ECs by 1) secretion of VEGFR2 via autophagosome and exosome and 2) degradation of VEGFR2 in autolysosome, which is coordinated with transcriptional suppression of VEGFR2 mRNAs by NOTCH signalling (see similar expression patterns of ADAM17 and γ-secretases to activate NOTCH signalling and autophagy-related genes in Fig. 4D).

**Fig. 6:**
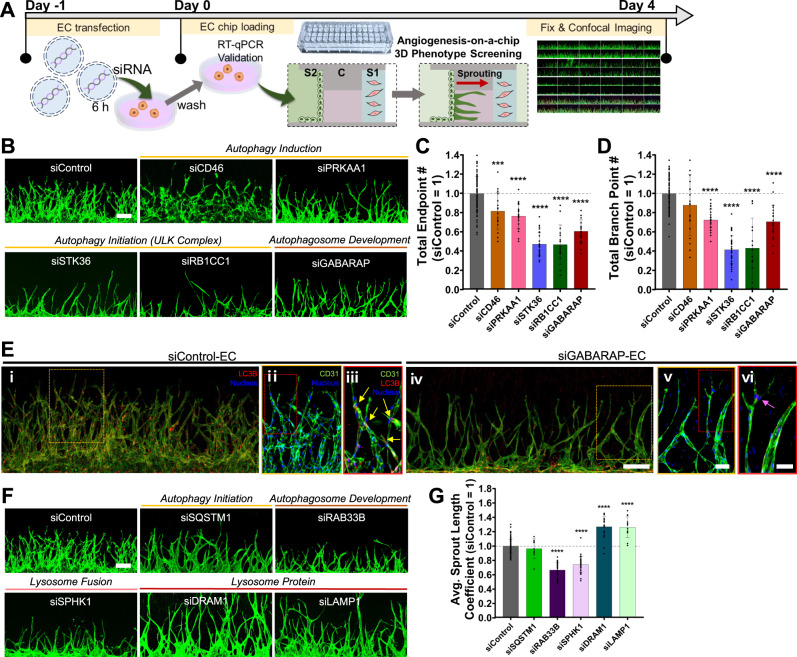
Reduced angiogenic sprouting by knockdown of autophagy-related genes activated in the basement or leading positions of elongating sprouts. **A** Schematic overview of sprouting experiments using siRNA-transfected ECs in 3D angiogenesis-on-a-chip. **B** Representative z-projected confocal images of angiogenic sprouts generated at day 4 from ECs transfected with the indicated siRNAs for the genes predominantly upregulated in the leading positions (Fig. 5B). Green, immunostaining with anti-CD31; and scale bar, 200 μm. **C**, **D** Quantitative analysis of endpoint and branch point counts of angiogenic sprouts generated from ECs transfected with the indicated siRNAs. ***p < 0.001, ****p < 0.0001, One-way ANOVA with Tukey’s posthoc test. *n* = 99 for siControl-transfected ECs; *n* = 23 for siPRKAA1- and siGABARAP-transfected ECs; *n* = 39, 20, 17 for siSTK36-, siCD46-, siRB1CC1-transfected ECs, respectively. Biological replicates. Data are mean ± S.D. Source data and exact *p*-values are provided in a Source Data file. **E** Representative z-projected 3D confocal image of angiogenic sprouts generated at day 4 from ECs transfected with either siControl or siGABARAP. Immunostainings with anti-CD31 (green), anti-LC3B (red), and Nucleus (blue) are shown. The areas (i and iv) indicated by yellow boxes (Scale bar, 200 μm) are magnified in ii and v (Scale bar, 100 μm), subregions of which (red boxes) are further magnified in iii and vi (Scale bar, 50 μm; yellow and pink arrowhead, LC3B puncta). Two times of experiments was repeated independently with similar results. **F** Representative z-projected confocal images of angiogenic sprouts generated at day 4 from ECs transfected with the indicated siRNAs for the genes predominantly upregulated in the basement positions (Fig. 5B and Supplementary Fig. 7). Scale bar, 200 μm. **G** Quantitative analysis on the length of angiogenic sprouts generated from ECs transfected with the indicated siRNAs. ****p < 0.0001, One-way ANOVA with Tukey’s posthoc test. *n* = 99 for siControl-transfected ECs; *n* = 34 for siRAB33B- and siSPHK1-transfected ECs; *n* = 21, 13, 9 for siDRAM1-, siLAMP1-, siSQSTM1-transfected ECs, respectively. Biological replicates. Data are mean ± S.D. Source data and exact *p*-values are provided in a Source Data file.

To test the hypothesis, we selected 5 autophagy-related genes predominantly activated in the leading position from the network model (Fig. 5B, bold labeled red nodes), knocked down each gene by loading HUVECs transfected with siRNAs targeting the gene to Channel S2 in our microfluidic organ-on-a-chip system (Fig. 6A and Supplementary Fig. 8A), and then obtained the confocal images at day 4 after loading of siRNA-transfected ECs (Fig. 6B). How these genes were selected and their associations genes with autophagy are summarized in Supplementary Fig. 8B and Table 2, respectively. To evaluate the knockdown effect, we computed the endpoint and branching points of the sprouts that can reflect the number of tip cells and tip cell selection in branching points, respectively. According to the hypothesis, knockdown of these genes is expected to reduce autophagy-dependent downregulation of VEGFR2 proteins, which can dysregulate tip-stalk cell specification and thus reduce the number of endpoints and branching points. Knockdown of the autophagy-related genes resulted in the decreases in the number of endpoints and branching points (Fig. 6C, D). For example, GABARAP knockdown led to reduction of the number of endpoints and branch points (Fig. 6E, i, ii, iv, and v). Correspondingly, the number of LC3B puncta was reduced near sprout endpoints in GABARAP knocked down condition (Fig. 6E, iii and vi). Interestingly, knockdown of CD46 and PRKAA1 that induce autophagy at early stage led to relatively weaker reductions in the number of endpoints and branching points (Fig. 6D, E), compared to that of the other genes associated with formation of membrane nucleation (STK36 and RB1CC1 in ULK complex) and phagophore (GABARAP) at later stages (Fig. 5B). Of note, knockdown of the genes led to similar changes in the length of sprouts to those in the endpoint and branch point counts, suggesting that the decreased endpoints can also affect sprout elongation (Supplementary Fig. 8C). These data support that the autophagy specifically activated in the stalk-like ECs may play important roles in tip-stalk cell specification during angiogenic sprouting.

The network model for autophagic activation in the basement of sprouts also suggests another hypothesis that autophagy can modulate cell cycle and apoptosis in proliferating ECs involved in sprout elongation (Fig. 5B). To test this hypothesis, we selected 5 autophagy-related genes predominantly activated in the sprout basement from the network model (Fig. 5B, bold labeled red nodes; Supplementary Fig. 7 and Table 2) and repeated the experiments after knocking down each of these genes (Supplementary Fig. 8D). To evaluate the knockdown effect, we mainly focused on the length of the sprouts that can reflect sprout elongation. According to the hypothesis, knockdown of these genes is expected to reduce autophagy-dependent quality control of proliferating ECs, which can dysregulate sprout elongation and thus reduce the length of the sprouts. Knockdown of these genes also led to the decreased length of the sprout (Fig. 6F, G), suggesting that the autophagy-dependent quality control of proliferating ECs is important for sprout elongation. Interestingly, as in the leading position, knockdown of SQSTM1 involved in the initiation of autophagy led to relatively weaker reductions in the sprout length (Fig. 6F, G), compared to that of 1) other genes associated with phagophore formation (RAB33B) and autophagosome-lysosome fusion (SPHK1) at later stages (Fig. 5B). These data support that the autophagy specifically activated in proliferating ECs may also play key roles in sprout elongation during angiogenic sprouting.

In contrast, interestingly, knockdown of the genes encoding lysosomal proteins (DRAM1 and LAMP1) led to the increase in the sprout length, suggesting that these genes have other lysosome-related functions than autophagy, and depletion of these functions may cause the increase in sprout length^47,48^. For example, lysosomes were regarded as suicide bags that would release unspecific digestive enzymes, such as cathepsins, which act as mediators of apoptosis^49^. Of note, knockdown of the genes led to similar changes in sprout endpoint and branch point counts to those in sprout length, suggesting close links among these morphological features (Supplementary Fig. 8E). Taken together, all these results demonstrate two modes of autophagy along elongating sprouts that might be associated with tip-stalk cell specification in the leading position and sprout elongation in the sprout basement and that both the modes are important for angiogenic sprouts.

## Discussion

The absence of proper experimental tools to recapitulate 3D angiogenic sprouting has hampered effective investigation of autophagic heterogeneity in ECs along elongating sprouts. In this study, scRNA-seq analysis of ECs along elongating sprouts in the 3D microfluidic angiogenesis-on-a-chip revealed two spatially differential activation of autophagy in the basement and leading positions of the sprouts, which was predicted to be associated with quality control of proliferating ECs for sprout elongation and tip-stalk cell specification, respectively. The two spatially differential activation modes of autophagy may change strategies for developing and optimizing autophagy-based anti-angiogenic therapeutics. Various microfluidics-based organ-on-a-chips or bead sprouting models mimicking human angiogenic sprouting have been reported for more than a decade^18,23,50–53^. These chips and models have been used for screening transient phenotypic changes after drug treatment or genetic modification. Moreover, these models are technically possible to be used for the type of single-cell analysis in this study. However, due to their limited reproducibility and throughput in generation of angiogenic sprouting, the spatially differential activation modes of autophagy might have not been effectively found through scRNA-seq analysis using these conventional models^54^. Our 3D angiogenesis-on-a-chip recently developed^24,25^ provides reproducible production of long elongated sprouts during the 3D long-term culture, owing to stable maintenance of cellular microenvironment, enabling to monitor non-transient systematic phenotypic changes of the sprouts including tip-stalk cell selection/specification, sprout elongation, and sprout branching^9,10,55^. Moreover, our system can be further modified to extend its application scope. For example, the long-term culture condition in our system can be further modified to mimic the varying local microenvironments (e.g., perturbation of local VEGF concentration gradients), allowing us to investigate the tip-stalk shuffling driven by continuous stochastic phenotype transition between tip and stalk cells under the varying local environments^39,55,56^. Furthermore, our system has the high-throughput capability to generate plenty of angiogenic sprouts from dozens of samples under multiple conditions (e.g., conditions treated with various autophagy inhibitors) in a single experimental setting, enabling high-content screening of drugs or genes associated with a particular angiogenic phenotype.

However, our system also has several limitations. In our system, HUVEC migrates into a fibrin gel, which does not reflect the physiological context of angiogenesis in vivo. Also, our system was optimized using HUVEC. Whether HUVEC or other primary ECs should be used in the in vitro angiogenesis assays has been extensively reviewed^57,58^. However, our results (Supplementary Fig. 6) have demonstrated that the major findings from our 3D chip are valid in vivo in human tumor tissues, supporting the utility of our chip despite the limitations. Moreover, our 3D chip can be also applicable to other primary ECs although the experimental settings (e.g., fibroblast type and concentrations of fibroblasts and ECs) should be modified. Finally, our system requires co-culture with fibroblasts, so do the previous systems^23,59–61^. Accordingly, perturbation of the system can affect not only ECs, but also fibroblasts, which makes it difficult to sort out whether the observed changes is driven by the direct effect on ECs or the indirect effect on ECs via fibroblasts. For example, CQ treatment reduced the number of fibroblasts at the boundary between Channels C and S1 (Supplementary Fig. 9A), which might decrease secretion of pro-angiogenic factors and reduce angiogenic sprouting. Nonetheless, plenty of fibroblasts (only ~12 and 22% decreases for 2 and 10 μM CQ, respectively) still remain to generate sufficient amounts of pro-angiogenic factors. We further observed the reduced angiogenic sprouts by *siATG5* or *siATG7* knockdown, consistent to those observed after chemical inhibitions, supporting that the reduced sprouting is likely due to direct effects of autophagy inhibition on ECs. However, further detailed experiments should be carried out for addressing the effect of the CQ-driven reduced fibroblast viability on sprouting ECs.

We presented an analytical tool for effectively evaluating morphological features of angiogenic sprouts, such as sprout length, width, endpoint count, and branch point count, from the 3D confocal images in our advanced culture system. Since these morphological features can be regulated differentially by diverse stress mechanisms^62^, such detailed morphological feature profiles could provide a clue for what morphological features of angiogenic sprouting autophagy regulates. For instance, knockdown of the autophagy-related genes highly upregulated in stalk-like ECs reduced the numbers of endpoints and branch points that can reflect the number of tip cells and tip cell selection at the branch points, respectively (Fig. 6B–D). These results imply potential association of autophagy with tip-stalk cell specification as predicted by the aforementioned model (Fig. 5B). On the other hand, when we estimated the morphological features, we used the z-projected 2D images converted from the original 3D confocal images to reduce computational loads for analyzing hundreds of the 3D images. However, the use of the 2D images is expected to result in the loss of information. For example, when a sprout branch point is identified, distinguishing whether a new sprout is being formed or two sprouts are simply crossing can be sometimes difficult in the z-projected 2D image. Although this problem was resolved partially during the skeletonization procedure by the Skeletonize function^63^ in the scikit-image processing package, there is still a possibility that the number of branch points could be overestimated. Nevertheless, the branch point counts predicted from this software well correlated with the numbers of branch points manually counted, and the increasing or decreasing patterns of branch points after the autophagy inhibitor treatment or knockdown of autophagy-related genes are expected to be still valid.

For the autophagic activation near sprout endpoints among the two activation modes, the reciprocal activation patterns of autophagy between tip- and stalk-like ECs suggest that the autophagic activation might contribute to tip-stalk specification. The proportion of stalk-like ECs in C1 was found to be approximately twice to that of tip-like ECs in C3 (Fig. 4, top heat map), which suggests that one tip-like EC leads the sprout toward the VEGF source and two stalk-like ECs trail the migrating tip-like EC, followed by the proliferating ECs (C2 and C4-6) to support sprout elongation. The predominant activation of autophagy in these two stalk-like ECs was tightly coordinated with the activation of NOTCH signaling that downregulates VEGFR2 mRNAs for the tip-stalk cell specification during the sprouting. This coordinated activation pattern, together with previously reported roles of autophagy, suggests a hypothesis that autophagy may contribute to downregulation of VEGFR2 proteins (Fig. 5B) to further solidify the stable tip-stalk cell specification under varying local environments. Moreover, among the two activation modes of autophagy, more autophagosomes were found in the basement, but their fusion to lysosome (i.e., autophagic flux) was more prevalent in the leading position. Further detailed functional experiments are needed to demonstrate why the autophagic flux is higher in the leading position. Specific knockdown of autophagy only in the stalk-like ECs highly expressing VEGFR1 and γ-secretase genes, or specific induction of autophagy in tip-like ECs highly expressing VEGFR2 and DLL4 can provide more thorough functional evaluation of autophagy in the specification of tip and stalk cells. The autophagy has been reported as an anti-angiogenic factor. An autophagy inducer, magnolol (Ery5), has an inhibitory effect on migration and tube formation properties in HUVECs while chemical blocking of autophagy by 3MA or gene silencing of ATG7 and LC3 reversed the anti-angiogenic effect of autophagy^12^. Opposite to the previous findings, however, both chemical blocking and gene silencing of autophagy-related genes led to significant reductions of angiogenic sprouting in our 3D microfluidic angiogenesis-on-a-chip. Compared to the conventional in vitro EC culture systems used in these previous studies, our advanced chip increases substantially EC proliferation and thus generates significantly larger numbers of angiogenic sprouts. Sprouting experiments using the ECs transfected with the mCherry-GFP tagged LC3B construct showed that the ratio of mCherry+ to GFP + LC3B expression was significantly decreased after CQ treatment compared to in the non-treated condition (Supplementary Fig. 9B–D), indicating the increased accumulation of autophagosome, but the decreased fusion of autophagosome and lysosome. These data support the efficacy of CQ in autophagy inhibition. However, the total number of sprouts (Fig. 2C) was significantly decreased after CQ treatment due to the reduced EC survival induced by autophagy inhibition^36,44^, as indicated by the reduced sprout length after CQ treatment (Fig. 2D). Accordingly, the subsets of sprouts expressing LC3B puncta (Fig. 2G) and GFP+ or mCherry+ LC3B puncta (Supplementary Fig. 9C) were also decreased. These data suggest that the reduced EC survival might be not significant in ECs with the low proliferation and sprouting potential in conventional in vitro EC culture systems, but become significant for highly sprouting ECs in our culture system. The same reasoning can be applied to the reduction in angiogenic sprouting after knockdown of autophagy-related genes because the knockdown can also reduce the survival potential in highly sprouting ECs.

We found that two different sets of genes were upregulated to activate two distinct modes of autophagy in the basement and leading positions of elongating sprouts. The two autophagic modes appear to be defined based on different cellular microenvironments, such as stalk-like ECs neighboring tip-like ECs or proliferating ECs with damaged substances from high levels of cell cycle and glycolysis. Autophagy is a single pathway undergoing from the initiation to sequential formation of phagophore, autophagosome, and autolysosome. Accordingly, our findings suggest that cells might upregulate different sets of genes to activate distinct modes of autophagy in response to cellular microenvironments. For example, autophagy modulates cell fate decisions during lineage commitment^64^, and also regulates proliferation of various cell types^65^. It would be interesting to investigate whether the two gene sets identified in this study could act as the shared molecular switches to differentially regulate these processes in other cell types under distinct microenvironments. Moreover, the reciprocal expression pattern of autophagy-related genes between stalk- and tip-like ECs may be able to shed insights into the controversial roles of autophagy in angiogenesis^7,8,66^. According to the reciprocal expression patterns, the induction of autophagy in tip cells having low expression of autophagy-related genes can lead to loss of their tip-like characteristics, which may result in reduced number of tip cells and lower sprouting, as seen in inhibition of JAG1 that suppresses activation of NOTCH signaling in tip cells^67^. In contrast, the autophagy induction in stalk cells having high expression of autophagy-related genes can solidify its stalk-like characteristics and tip-stalk cell specification, which may promote angiogenic sprouts. Taken together, these data suggest the need for a strategy to differentially target stalk and tip cells when developing autophagy-based anti-angiogenic therapeutics.

## Methods

### Cell culture

Human umbilical vein endothelial cells (HUVECs) and human lung fibroblasts (LFs) were purchased from Lonza (C2519A for HUVEC and CC-2512 for LF). HUVECs were cultured in endothelial growth medium-2 (EGM-2) with full supplements (FBS, hydrocortisone, hFGF-B, VEGF, R3-IGF-1, ascorbic acid, hEGF, GA-1000, and heparin) included in the product (Lonza, CC-3162). Normal LFs were cultured in fibroblast growth medium-2 (FGM-2, Lonza, CC-3131) with full supplements. HUVECs and LFs were used at passage numbers 4–5 and 6–7, respectively. Cells were grown no more than 80% of confluency in dish prior to passaging or loading on the chip.

### Fabrication of microfluidic high-throughput cell culture chip

The chip for 3D cell culture was made with polystyrene (PS) and injection molding method was used for the fabrication by R&D Factory (Korea). The aluminum alloy mold core for injection molding was made through machining and polishing. The setting for injection molding was clamping force of 130 ton, maximum injection pressure of 55 bar, cycle time of 15 s, and nozzle temperature 200°C. The plastic product was bonded with a pressure-sensitive adhesive-coated polycarbonate film which function as the bottom of the culture chip.

### 3D angiogenesis assay using angiogenesis-on-a-chip

For 3D angiogenic sprouting assay, bovine fibrinogen (Sigma, F8630) was dissolved with warmed phosphate-buffered saline (PBS, Hyclone, SH30256.01) for 30 min without vortexing to generate fibrin solution in concentration of 10 mg mL^–1^. The solution was then mixed with aprotinin solution (0.15 U mL^–1^, Sigma, A1153) in volume ratio of 25: 4, to avoid degradation and instability of fibrin hydrogel during long-term 3D angiogenic sprouting culture^68^. In all cases, acellular hydrogel patterning on microfluidic channel was done by mixing the fibrin solution with PBS to generate final concentration of 4 mg mL^–1^. On the other hand, patterning cellular hydrogel containing fibroblasts was performed by mixing the hydrogel solution and fibroblast cell suspension to generate final concentration of 2.5 mg mL^–1^ and 7.5 × 10^6^ cells mL^–1^, respectively. For 3D hydrogel loading on every channel, after the fibrin solution with or without cells are mixed, 1 μL of thrombin solution (0.5 U mL^–1^, Sigma, T4648) was added with short gentle pipetting and then immediately introduced into the channel. Regarding the order to load the channels in each chip, acellular hydrogel was first loaded on Channel C with volume of 0.9 μL per channel; cellular hydrogel with fibroblasts was then loaded on Channel S1 in volume of 3 μL per each channel in 10 min after the hydrogel in Channel C was crosslinked; and in 5 min after Channel S1, HUVEC suspension in concentration of 3 × 10^6^ cells mL^–1^ was finally loaded on Channel S2. The chip is then vertically set for 30 min in incubator in order to make the HUVECs to attach on the side wall of hydrogel patterning on Channel C. Finally, the chip is filled with cell culture media and incubated at 37 °C in 5% CO_2_. All liquid patterning of hydrogel or cell suspension is done based on the mechanism of capillary flow-driven robust patterning introduced in previous work^26^. To promote angiogenic sprouting, the interstitial flow was given in all samples by generating hydrostatic pressure based on difference in media level between two reservoirs in each well^18^. After 3–4 days of angiogenic sprouting assay, samples were fixed using 4% paraformaldehyde (Biosesang, PC2031-050-00) for 20 min and then replaced with PBS for long-term preservation in 4 °C.

### Drug treatment

Autophagy inhibitors were prepared in stock concentration according to the manufacturer’s instructions: chloroquine diphosphate (Sigma-Aldrich, C6628), wortmannin (Sigma-Aldrich, W1628), 3-methyladenine (3MA, Cayman, 13242), and bafilomycin A1 (Cayman, 11038). The inhibitors were then diluted into each working concentration with cell culture medium and applied into media reservoir a day or two days after EC loading. Two types of control medium were set considering the addition of DMSO as solvent for wortmannin, 3MA, and bafilomycin A1; control medium (EGM-2) and 0.5% DMSO control medium.

### Transfection of siRNA or LC3B-mCherry-GFP dual reporter

Autophagy-related genes were first selected from the genes in P1 and 5 based on the following criteria: 1) genes annotated with autophagy-related terms according to KEGG (autophagy pathway), WikiPathway (autophagy pathway), GO Biological Process (autophagy and autophagosome organization), and Reactome (autophagy and macro-autophagy); and 2) genes whose associations with autophagy were previously reported in the literature. This annotation information and previous literature are summarized in Supplementary Table 2. For each of the selected genes, lipofectamineTM 3000 transfection reagent kit (Invitrogen, L3000001) was used according to the manufacturer’s protocol to transfect siRNA targeting the gene to HUVECs. The sequences of each autophagy-related genes are listed in Supplementary Table 3. Lipofectamine 3000-siRNA complex was initially generated in volume ratio of 3: 1 and mixed on endothelial basal medium (EBM, Lonza, CC-3156) and incubated for 20 min at room temperature. For transfection of LC3B-mCherry-GFP dual reporter plasmid (pLVX-mCherry-GFP-hLC3B) to HUVECs, lipofectamine 3000-P3000- plasmid complex was initially generated in volume ratio of 2: 2: 1, followed by the mixing and incubation as described above. Either of the mixture was then added onto HUVECs in culture dish in confluency of 70–80% for 6 h in final siRNA concentration of 20 nM or plasmid concentration of 100 ng/mL. The Lipofectamine complex was washed out after 6 h with fresh media and culture overnight. HUVECs were then detached from the dish and loaded on the microfluidic chip for 3D angiogenesis assay.

### RNA isolation from ECs and quantitative real-time PCR (qRT-PCR)

Total RNA isolation from HUVECs treated with different types of siRNA for 12 h was conducted using Trizol reagent (Invitrogen, 15596026) by following the instruction from the manufacturer. First-strand cDNA from extracted total RNA was synthesized using SuperScript^TM^III First-Strand Synthesis System (Invitrogen, 18080051) by following the instructions from the manufacturer. Quantitative RT-PCR (qRT-PCR) analysis was performed in StepOnePlus Real-Time PCR System (Applied Biosystems) using TB Green Premix Ex Taq^TM^II(Takara, RR82WR). Primers for qRT-PCR analysis were listed in Supplementary Table 4.

### Immunofluorescence staining and confocal imaging

Fixed samples cultured in angiogenesis-on-a-chip were permeabilized using 0.2% Triton-X 100 (Sigma-Aldrich) for 20 min, then treated with 3% bovine serum albumin (Sigma-Aldrich) for 40 min to prevent unspecific binding of immunofluorescent antibodies. Endothelial cells are labelled with either antibody or lectin. Alex Flour 488- or 594-conjugated mouse anti-human cluster of differentiation 31 (anti-CD31, Biolegend, 303110 and 303126, Clone: WM59) was treated in 1:200 ratio for 3 days. Two types of lectin, fluorescein-conjugated Ulex Europaeus Agglutinin 1 (Vector, FL-1061) and DyLight 594-conjugated Ulex Europaeus Agglutinin 1 (Vector, DL-1067) were treated in 1:1000 ratio for 3 days. To label autophagosome, rabbit polyclonal anti-human LC3B antibody (Abcam, ab48394) was treated in 1:200 ratio for 3 days, then the samples were subsequently incubated with Alex Fluor 568 goat polycloncal anti-rabbit immunoglobulin G (IgG, Invitrogen, A-11036) in 1:500 ratio as secondary antibody overnight. Hoechst 33342 (Molecular Probes, H3570) was used to stain DNA by incubating samples in 1000:1 ratio overnight. All fluorescence molecules were diluted in BSA. Samples were incubated in PBS after fluorescence staining and preserved in 4°C before imaging. All 3D fluorescent imaging was conducted using confocal microscopy Nikon Eclipse Ti2-E (Nikon).

### Image processing and quantitative analysis on morphology of angiogenic sprouts

Fiji (http://fiji.sc.), an open-access software, was used to process confocal images in advance of quantitative analysis. 3D confocal images were stacked and converted to 2D images using z-projection in max intensity. Images were cropped in the equal region of interest. Multiple and consistent pre-processing methods were used to denoise each image, such as averaging filtering, median filtering, and Gaussian filtering. As Gaussian filtering could preserve the value of the original shape of sprouts better than other methods, the Gaussian filter was first adapted to denoise images. Then we developed a small-blob-remove algorithm process by using python OpenCV to denoise small blobs made from Gaussian filter and dead cell debris. After denoising, skeletonization was conducted with an equal threshold value for every image in a single set of analysis. We also developed an analytic algorithm for quantifying morphological features based on skeletonization images. By using this algorithm, the total pixel number of the masked image was quantified and represented as sprout area, and the total pixel number of the skeletonized image was defined as the total length of the sprouts within a sample. In order to count the total sprout number within a sample, we first recorded the coordinates of each endpoint in the skeletonized sprout. Since there were lots of endpoints around the roots of the sprouts, which affects the average of y-coordinates and interrupts quantification of images reflecting morphological features, we then established the criterion to only record skeleton endpoint which has its y-coordinates higher than the median value within each sample and applied this criterion to all samples in the analysis. We counted the recorded number of skeleton endpoints and represented it as the total endpoint number of sprouts, and the average of y-coordinate values was defined as the average sprout length coefficient. The total sprout area was divided by the total length of the skeletonized sprout and was represented as the average sprout width coefficient for each sample. The number of branch points was represented as the total junction number.

### Sample Collection from angiogenesis-on-a-chip for single-cell RNA sequencing

Based on the protocol from Carrion et al.’s work^69^, the nattokinase powder was dissolved in the PBS at a concentration of 100 FU and was warmed enough in 37 °C water bath. The solution was centrifuged at 400 *g* for 5 min and filtered with the 0.2 μm pore-sized filter before treatment in the microfluidic chip. After washing the samples in the microfluidic chip with PBS, the nattokinase solution was treated directly to fibrin hydrogel through the medium reservoir for 20 min. Completely detached cells were harvested from the microfluidic chip and stored in EGM-2 at 4°C, which were immediately processed for scRNA-seq analysis.

### Single-cell RNA sequencing

Libraries for scRNA-seq were generated using Chromium Single Cell 3’ Library & Gel Bead Kit v2 (10X Genomics, PN-120237) and Chromium Single cell A Chip Kit (10X Genomics, PN-120236), and Chromium i7 Multiplex Kit (10X Genomics, PN-120262). All the processes were performed according to the manufacture’s protocol. Briefly, cells were counted and diluted to 4 × 10^5^ ~ 2 × 10^6^/ml concentration. The cell suspension was added to the reverse transcription reaction mixture and loaded onto A Chip aiming for capture of 3000 cells. Gel Bead-In-Emulsions (GEMs) were generated using Chromium Controller. GEMs were incubated in a thermal cycler for reverse transcription. After cDNA recovery from GEMs, cDNA was amplified using a thermal cycler. The size and quantity of amplified cDNA were analyzed using Bioanalyzer (Agilent). Subsequent procedures including enzymatic fragmentation, end repair, A-tailing, adaptor ligation, and sample index PCR for the sequencing library were performed using the purified cDNA. The quality of the final library was analyzed using Bioanalyzer High Sensitivity Chip (Agilent). Libraries were pooled and sequenced through the Hiseq2500 platform (Illumina) with the following parameters: Read1: 26 cycles, i7 index: 8 cycles, i5 index: 0 cycles, and Read2: 98 cycles.

### Data analysis in single-cell RNA sequencing

#### Pre-processing and quality control

Raw FASTQ files were processed with the Cell Ranger software (v.2.0.0) using default arguments. Reads were aligned to the human reference genome (GRCh38) with the Ensembl GRCh38.89 annotation. A gene-by-cell unique molecular identifier (UMI) count matrix for both conditions was generated and aggregated into a single count matrix with “expect-cells = 3000”. We then filtered out cells that correspond to empty droplets after defining a threshold in the barcode rank plots. We removed low-quality cells that have greater than 10% UMIs assigned to mitochondrial encoded genes or lower than 1000 total UMI counts. The thresholds were chosen by visually inspecting outliers in the principal component analysis (PCA) plot on the quality control metrics using the scater (v.1.22.0) R package^70^.

#### Normalization and highly variable genes selection

The raw count matrix was normalized by cell-specific size factors estimated by the scran (v.1.22.1) R package^71^, and then log2-transformed with a pseudo-count of 1. Highly variable genes (HVGs) were identified using the same package with (FDR) ≤ 0.05.

#### Cell sampling for batch correction

The number of the profiled ECs substantially differs between two conditions for which independent scRNA-seq analyses were performed- 2372 cells in the condition with fibroblasts and 12,186 cells in the condition without fibroblasts. To balance the number of ECs between the two datasets for effective batch correction and the subsequent analyses, a subset of ECs in the condition without fibroblasts were sampled to be comparable to the number of ECs in the condition with fibroblasts. All ECs in the condition without fibroblasts were first clustered using the FindClusters function in Seurat (v.4.3.0)^72^ with the first 20 PCs (k.param = 10 and resolution = 0.5). To preserve the transcriptomic characteristics of the data, 250 cells were sampled from each cluster, yielding a total of 2321 ECs from the condition without fibroblasts. These 2321 ECs were then integrated with 2372 cells from the condition with fibroblasts for batch correction, clustering, and trajectory analysis.

#### Batch effect correction

To correct batch effects between the two datasets generated with fibroblasts and without fibroblasts, we first used the FindVariableFeatures function of the Seurat to obtain 2000 highly variable genes (HVGs) from each dataset. The two datasets were then anchored using the FindIntegrationAnchors function with the first 10 PCs. The two conditions were finally integrated using the IntegrateData function with the first 10 PCs.

#### Cell cycle correction

Cell cycle effects were corrected using the ScaleData function of the Seurat by setting vars.to.regress to the S phase and G2M phase score due to the high cell cycle dependency. Both S and G2M phase scores were calculated by the CellCycleScoring function of the same package.

#### Visualization and clustering

All cells were visualized in the uniform manifold approximation and projection (UMAP) plot using the RunUMAP function of the Seurat with the first 30 PCs. Cells were clustered by the FindClusters function of the same package with the first 30 PCs with default parameters. Low-quality clusters with a low UMI count and a low number of detected genes were removed. Cells were then visualized by the RunUMAP with default parameters. Cells were clustered using the FindClusters function using the first 30 PCs with default parameters.

#### Signature scores

Gene set signature scores of each cell were computed using the AddModuleScore of the Seurat with the glycolysis gene set of MSigDB, and the marker gene sets for tip- and stalk-like cells^39^, and the scores were then z-scaled and averaged across each cluster.

#### Trajectory analysis

The monocle3(v.1.3.1) R package was used for the trajectory analysis. The batch-corrected gene expression matrix containing 2000 HVGs was processed by preprocess_cds of the same packages with the first 10 PCs. Cell cycle effects were removed using the align_cds function. Cells were clustered using the cluster_cells function with a resolution of 0.001. The principal graph was then fit using the learn_graph function. The relative order of cells along the trajectory was calculated using the order_cells function.

#### Identification of dynamic patterns along the trajectory

We ordered cells along the trajectory using the relative order of cells obtained from the order cells function of the Monocle3(v.1.3.1) R package. We selected the highly variable genes (HVGs) and the union of the marker genes in each cluster as a set of feature genes. We then performed clustering of the feature genes based on their smoothed expression profiles along the trajectory using a hierarchical clustering method (Euclidean distance as a dissimilarity measure and ward linkage method). We identified initially 17 clusters from the hierarchical clustering. From these initial clusters, we finally defined four expression patterns (P1-4) by merging them with the similar dynamic expression patterns along the trajectory after removing small clusters (<30 genes, which corresponded to ~2% of the total number of genes used). Moreover, we also removed the clusters showing expression patterns inconsistent to the major patterns identified when the trajectory analysis was done only using ECs in the presence of fibroblasts, considering that they might be potential artifacts produced when the datasets from the two conditions were merged. For each gene, the smoothed relative gene expression profile along each of the trajectories was plotted using MATLAB (plot.m in R2019a; www.mathworks.com/) in a 50-cell interval for the trajectory.

### The data analysis of publicly available breast cancer EC data

We used a scRNA-seq dataset of tumoral human breast tissue^41^. Raw counts and the metadata were downloaded from the gene expression omnibus database (GEO), under accession code GSE15510941. The data analysis of breast cancer EC data was performed as described for processing of the HUVEC scRNA-seq dataset. After clustering ECs into 12 clusters annotated by transferring cell types from the original study, we removed artery i/ii and lymphatic clusters not involved in angiogenesis and breast-specific capillary ii cluster. After categorizing the remaining clusters into proliferating, stalk-like, and tip-like EC clusters as described above, we then performed trajectory analysis twice using the following sets of EC clusters: (1) proliferating and stalk-like EC clusters + angiogenic LS cluster and (2) proliferating and stalk-like EC clusters + angiogenic cluster.

### Functional enrichment and network analysis

Functional enrichment analysis of the genes with early, middle, or late upregulation along the trajectories was performed using ConsensusPathDB software (version 35)^40^. Gene ontology biological processes (GOBPs) and pathways enriched by the genes were identified as the ones with *P* < 0.05. The network model was built using protein-protein interactions and activation/suppression information obtained from the Kyoto Encyclopedia of Genes and Genomes (KEGG) pathway database^73^ for the genes involved in the representative pathways enriched by the three sets of the genes (early, middle, or late upregulated). The resulting network was visualized using Cytoscape (version 3.8.2)^74^, and the nodes in the network model were arranged based on their localizations in their associated pathways with the edges indicating activation/inhibition information from the KEGG pathway database.

### Statistical analysis

All statistical analyses were performed with GraphPad Prism 9 software. We used unpaired t-test and one-way ANOVA with subsequent Tukey’s multiple comparisons post-test depending on the experimental paradigm. *P* < 0.05 was considered significant (*p < 0.05, **p < 0.01, ***p < 0.001, and ****p < 0.0001; n.s., not significant; n.d., not detected). Data are expressed as the mean ± S.D. and *n* values are stated in figure legends.

### Reporting summary

Further information on research design is available in the Nature Portfolio Reporting Summary linked to this article.

### Supplementary information

Supplementary Information

Description of additional supplementary files

Supplementary Data 1

Reporting Summary

### Source data

Source data

## Data Availability

The Single-cell transcriptomic data generated in this study have been deposited in the Sequence Read Archive (SRA) database under accession code PRJNA931762. The publicly available breast cancer EC data used in this study are available in the gene expression omnibus database (GEO) database under accession code GSE155109. Other data generated in this study are provided in the Supplementary Information and Source Data file. Any additional requests for information can be directed to, and will be fulfilled by, the corresponding authors. Source data are provided with this paper.

## Code availability

The R scripts used in this study are available at https://github.com/CB-postech/scRNA-HUVECs/. Also the codes have been deposited to Zenodo [10.5281/zenodo.10280004]^75^.

## References

[1] Levine B, Klionsky DJ (2004). Development by self-digestion: molecular mechanisms and biological functions of autophagy. Dev. Cell.

[2] Maiuri MC, Zalckvar E, Kimchi A, Kroemer G (2007). Self-eating and self-killing: crosstalk between autophagy and apoptosis. Nat. Rev. Mol. Cell Biol..

[3] Mizushima N (2007). Autophagy: process and function. Genes Dev..

[4] Du J-H (2017). Role of autophagy in angiogenesis induced by a high-glucose condition in RF/6A cells. Ophthalmologica.

[5] Li R, Du J, Chang Y (2016). Role of autophagy in hypoxia-induced angiogenesis of RF/6A cells in vitro. Curr. Eye Res..

[6] Liang P (2018). Autophagy promotes angiogenesis via AMPK/Akt/mTOR signaling during the recovery of heat-denatured endothelial cells. Cell Death Dis..

[7] Hassanpour M, Rezabakhsh A, Pezeshkian M, Rahbarghazi R, Nouri M (2018). Distinct role of autophagy on angiogenesis: highlights on the effect of autophagy in endothelial lineage and progenitor cells. Stem Cell Res. Ther..

[8] Schaaf MB, Houbaert D, Meçe O, Agostinis P (2019). Autophagy in endothelial cells and tumor angiogenesis. Cell Death Differ..

[9] Adams RH, Alitalo K (2007). Molecular regulation of angiogenesis and lymphangiogenesis. Nat. Rev. Mol. Cell Biol..

[10] Jakobsson L (2010). Endothelial cells dynamically compete for the tip cell position during angiogenic sprouting. Nat. Cell Biol..

[11] Yetkin-Arik B (2019). Endothelial tip cells in vitro are less glycolytic and have a more flexible response to metabolic stress than non-tip cells. Sci. Rep..

[12] Kumar S (2013). Autophagy triggered by magnolol derivative negatively regulates angiogenesis. Cell Death Dis..

[13] Sung SJ, Kim H-K, Hong Y-K, Joe YA (2019). Autophagy is a potential target for enhancing the anti-angiogenic effect of mebendazole in endothelial cells. Biomol. Ther..

[14] Nishikawa T (2010). The inhibition of autophagy potentiates anti-angiogenic effects of sulforaphane by inducing apoptosis. Angiogenesis.

[15] Zou J (2019). VEGF‐A promotes angiogenesis after acute myocardial infarction through increasing ROS production and enhancing ER stress‐mediated autophagy. J. Cell. Physiol..

[16] Zheng Y (2018). By activating Akt/eNOS bilobalide B inhibits autophagy and promotes angiogenesis following focal cerebral ischemia reperfusion. Cell. Physiol. Biochem..

[17] Torisu T (2013). Autophagy regulates endothelial cell processing, maturation and secretion of von Willebrand factor. Na. Med..

[18] Kim S, Chung M, Ahn J, Lee S, Jeon NL (2016). Interstitial flow regulates the angiogenic response and phenotype of endothelial cells in a 3D culture model. Lab. Chip.

[19] Sobrino A (2016). 3D microtumors in vitro supported by perfused vascular networks. Sci. Rep..

[20] Osaki T, Sivathanu V, Kamm RD (2018). Vascularized microfluidic organ-chips for drug screening, disease models and tissue engineering. Curr. Opin. Biotechnol..

[21] Lee S (2018). Microfluidic-based vascularized microphysiological systems. Lab Chip.

[22] Haase K, Kamm RD (2017). Advances in on-chip vascularization. Regen. Med..

[23] Kim S, Lee H, Chung M, Jeon NL (2013). Engineering of functional, perfusable 3D microvascular networks on a chip. Lab. Chip.

[24] Lee S (2021). Modeling 3D Human Tumor Lymphatic Vessel Network Using High‐throughput Platform. Adv. Biol..

[25] Yu J (2022). Perfusable micro-vascularized 3D tissue array for high-throughput vascular phenotypic screening. Nano Converg..

[26] Lee Y (2018). Microfluidics within a well: an injection-molded plastic array 3D culture platform. Lab. Chip.

[27] Lu H (2017). TFEB inhibits endothelial cell inflammation and reduces atherosclerosis. Sci. Signal..

[28] Li Z (2019). High concentration of sodium metasilicate impairs autophagic flux and induces apoptosis in human umbilical vein endothelial cells. Biol. Trace Elem. Res..

[29] Srisook K, Potiprasart K, Sarapusit S, Park C-S, Srisook E (2020). Etlingera pavieana extract attenuates TNF-α induced vascular adhesion molecule expression in human endothelial cells through NF-κB and Akt/JNK pathways. Inflammopharmacology.

[30] Gomes AM, Pinto TS, da Costa Fernandes CJ, da Silva RA, Zambuzzi WF (2020). Wortmannin targeting phosphatidylinositol 3‐kinase suppresses angiogenic factors in shear‐stressed endothelial cells. J. Cell. Physiol..

[31] Alhosin M (2013). Redox-sensitive up-regulation of eNOS by purple grape juice in endothelial cells: role of PI3-kinase/Akt, p38 MAPK, JNK, FoxO1 and FoxO3a. PLoS One.

[32] Eng CH (2016). Macroautophagy is dispensable for growth of KRAS mutant tumors and chloroquine efficacy. Proc. Natl Acad. Sci..

[33] Ye H (2016). Chloroquine, an autophagy inhibitor, potentiates the radiosensitivity of glioma initiating cells by inhibiting autophagy and activating apoptosis. BMC Neurol..

[34] Jeong I-H, Bae W-Y, Choi J-S, Jeong J-W (2020). Ischemia induces autophagy of endothelial cells and stimulates angiogenic effects in a hindlimb ischemia mouse model. Cell Death Dis..

[35] Wang Q, Liang B, Shirwany NA, Zou M-H (2011). 2-Deoxy-D-glucose treatment of endothelial cells induces autophagy by reactive oxygen species-mediated activation of the AMP-activated protein kinase. PloS One.

[36] Gregorio P (2021). Chloroquine may induce endothelial injury through lysosomal dysfunction and oxidative stress. Toxicol. Appl. Pharmacol..

[37] Collier JJ, Suomi F, Oláhová M, McWilliams TG, Taylor RW (2021). Emerging roles of ATG7 in human health and disease. EMBO Mol. Med..

[38] Collier JJ (2021). Developmental consequences of defective ATG7-mediated autophagy in humans. N. Engl. J. Med..

[39] Chen W (2019). The endothelial tip-stalk cell selection and shuffling during angiogenesis. J. Cell Commun. Signal..

[40] Kamburov A, Wierling C, Lehrach H, Herwig R (2009). ConsensusPathDB—a database for integrating human functional interaction networks. Nucleic Acids Res..

[41] Geldhof V (2022). Single cell atlas identifies lipid-processing and immunomodulatory endothelial cells in healthy and malignant breast. Nat. Commun..

[42] Uberti F (2014). Vitamin D protects human endothelial cells from oxidative stress through the autophagic and survival pathways. J. Clin. Endocrinol. Metab..

[43] Han J (2012). Curcumin induces autophagy to protect vascular endothelial cell survival from oxidative stress damage. Autophagy.

[44] Rezabakhsh A (2017). Rapamycin inhibits oxidative/nitrosative stress and enhances angiogenesis in high glucose-treated human umbilical vein endothelial cells: role of autophagy. Biomed. Pharmacother..

[45] Liu, H., Yu, S., Zhang, H. & Xu, J. Angiogenesis impairment in diabetes: role of methylglyoxal-induced receptor for advanced glycation endproducts, autophagy and vascular endothelial growth factor receptor 2. * PloS One* **4**, e46720 (2012). 10.1371/journal.pone.0046720 PMC3463541 23056421

[46] Atienzar‐Aroca S (2018). Role of retinal pigment epithelium‐derived exosomes and autophagy in new blood vessel formation. J. Cell. Mol. Med..

[47] Lyu J (2017). Pharmacological blockade of cholesterol trafficking by cepharanthine in endothelial cells suppresses angiogenesis and tumor growth. Cancer Lett..

[48] Tang T (2020). The role of lysosomes in cancer development and progression. Cell Biosci..

[49] Leist M, Jäättelä M (2001). Triggering of apoptosis by cathepsins. Cell Death Differ..

[50] Lee S (2020). 3D microfluidic platform and tumor vascular mapping for evaluating anti-angiogenic RNAi-based nanomedicine. ACS Nano.

[51] van Duinen V (2020). Robust and scalable angiogenesis assay of perfused 3D human iPSC-derived endothelium for anti-angiogenic drug screening. Int. J. Mol. Sci..

[52] Kim C, Kasuya J, Jeon J, Chung S, Kamm RD (2015). A quantitative microfluidic angiogenesis screen for studying anti-angiogenic therapeutic drugs. Lab. Chip.

[53] Schulz MMP (2012). Phenotype-based high-content chemical library screening identifies statins as inhibitors of in vivo lymphangiogenesis. Proc. Natl Acad. Sci..

[54] Goveia J (2020). An integrated gene expression landscape profiling approach to identify lung tumor endothelial cell heterogeneity and angiogenic candidates. Cancer Cell.

[55] Wood L, Kamm R, Asada H (2011). Stochastic modeling and identification of emergent behaviors of an Endothelial Cell population in angiogenic pattern formation. Int. J. Robot. Res..

[56] Venkatraman L, Regan ER, Bentley K (2016). Time to decide? Dynamical analysis predicts partial tip/stalk patterning states arise during angiogenesis. PLoS One.

[57] Medina-Leyte DJ, Domínguez-Pérez M, Mercado I, Villarreal-Molina MT, Jacobo-Albavera L (2020). Use of human umbilical vein endothelial cells (HUVEC) as a model to study cardiovascular disease: A review. Appl. Sci..

[58] Lee S, Chung M, Lee SR, Jeon NL (2020). 3D brain angiogenesis model to reconstitute functional human blood–brain barrier in vitro. Biotechnol. Bioeng..

[59] Newman AC, Nakatsu MN, Chou W, Gershon PD, Hughes CC (2011). The requirement for fibroblasts in angiogenesis: fibroblast-derived matrix proteins are essential for endothelial cell lumen formation. Mol. Biol. Cell.

[60] Nakatsu MN (2003). Angiogenic sprouting and capillary lumen formation modeled by human umbilical vein endothelial cells (HUVEC) in fibrin gels: the role of fibroblasts and Angiopoietin-1✩. Microvasc. Res..

[61] Sanchez B (2019). Impact of human dermal microvascular endothelial cells on primary dermal fibroblasts in response to inflammatory stress. Front. Cell Dev. Biol..

[62] Corliss BA, Mathews C, Doty R, Rohde G, Peirce SM (2019). Methods to label, image, and analyze the complex structural architectures of microvascular networks. Microcirculation.

[63] Zhang TY, Suen CY (1984). A fast parallel algorithm for thinning digital patterns. Commun. ACM.

[64] Sharma K (2022). Autophagy modulates cell fate decisions during lineage commitment. Autophagy.

[65] Mathiassen SG, De Zio D, Cecconi F (2017). Autophagy and the cell cycle: a complex landscape. Front. Oncol..

[66] Kardideh B, Samimi Z, Norooznezhad F, Kiani S, Mansouri K (2019). Autophagy, cancer and angiogenesis: where is the link?. Cell Biosci..

[67] Blanco R, Gerhardt H (2013). VEGF and Notch in tip and stalk cell selection. Cold Spring Harbor Perspect. Med..

[68] Lorentz KM, Kontos S, Frey P, Hubbell JA (2011). Engineered aprotinin for improved stability of fibrin biomaterials. Biomaterials.

[69] Carrion B, Janson IA, Kong YP, Putnam AJ (2014). A safe and efficient method to retrieve mesenchymal stem cells from three-dimensional fibrin gels. Tissue Eng. Part C: Methods.

[70] McCarthy DJ, Campbell KR, Lun AT, Wills QF (2017). Scater: pre-processing, quality control, normalization and visualization of single-cell RNA-seq data in R. Bioinformatics.

[71] L Lun AT, Bach K, Marioni JC (2016). Pooling across cells to normalize single-cell RNA sequencing data with many zero counts. Genome Biol..

[72] Butler A, Hoffman P, Smibert P, Papalexi E, Satija R (2018). Integrating single-cell transcriptomic data across different conditions, technologies, and species. Nat. Biotechnol..

[73] Kanehisa M, Goto S, Sato Y, Furumichi M, Tanabe M (2012). KEGG for integration and interpretation of large-scale molecular data sets. Nucleic Acids Res..

[74] Caporaso JG, Kuczynski J, Stombaugh J, Bittinger K, Knight R (2010). QIIME allows analysis of high-throughput community sequencing data. Nat. Methods.

[75] Kim B. S. & Che S. H., Angiogenesis-on-a-chip coupled with single-cell RNA sequencing reveals spatially differential activations of autophgy along angiogenic sprouts, CB-postech/scRNA-HUVECs: scRNA-HUVECs released, 10.5281/zenodo.10280003 (2023).10.1038/s41467-023-44427-038172108

