# Supplementary material for: Angiogenesis-on-a-chip coupled with single-cell RNA sequencing reveals spatially differential activations of autophagy along angiogenic sprouts
Source: Nat Commun. 2024 Jan 3;15:230. doi: 10.1038/s41467-023-44427-0 (PMC10764361; doi:10.1038/s41467-023-44427-0)
Supplement: Supplementary file 1 — Supplementary Information [file 41467_2023_44427_MOESM1_ESM.pdf]

Supplementary Information

Angiogenesis-on-a-chip coupled with single-cell RNA sequencing reveals spatially differential activations of autophagy along angiogenic sprouts

Somin Lee[1,2,12], Hyunkyung Kim[3,4,12], Bum Suk Kim[5,12], Sehyun Chae[6,12], Sangmin Jung[7,12], Jung Seub Lee[7], James Yu[1], Kyungmin Son[7], Minhwan Chung[7], Jong Kyoung Kim[*5,8], Daehee Hwang[*9], Sung Hee Baek[*10], Noo Li Jeon[*1,2,7,11]

[1]Interdisciplinary Program for Bioengineering, Seoul National University, Seoul, South Korea.
[2]Institute of Advanced Machines and Design, Seoul National University, Seoul, South Korea.
[3]Department of Biochemistry and Molecular Biology, Korea University College of Medicine, Seoul, South Korea.
[4]BK21 Graduate Program, Department of Biomedical Sciences, Korea University College of Medicine, Seoul, South Korea.
[5]Department of New Biology, DGIST, Daegu, South Korea.
[6]Neurovascular Unit Research Group, Korea Brain Research Institute, Daegu, South Korea.
[7]Department of Mechanical Engineering, Seoul National University, Seoul, South Korea.
[8]Department of Life Sciences, Pohang University of Science and Technology (POSTECH), Pohang, South Korea.
[9]School of Biological Science, Seoul National University, Seoul, South Korea.
[10]Creative Research Initiatives Center for Epigenetic Code and Diseases, School of Biological Sciences, Seoul National University, Seoul, South Korea.
[11]Qureator, Inc., San Diego, CA, USA
[12]These authors contributed equally to this work
[*]Corresponding authors
E-mail: njeon@snu.ac.kr (N. L. J.); sbaek@snu.ac.kr (S. H. B.); daehee@snu.ac.kr (D. H.); blkimjk@postech.ac.kr (J. K. K.)

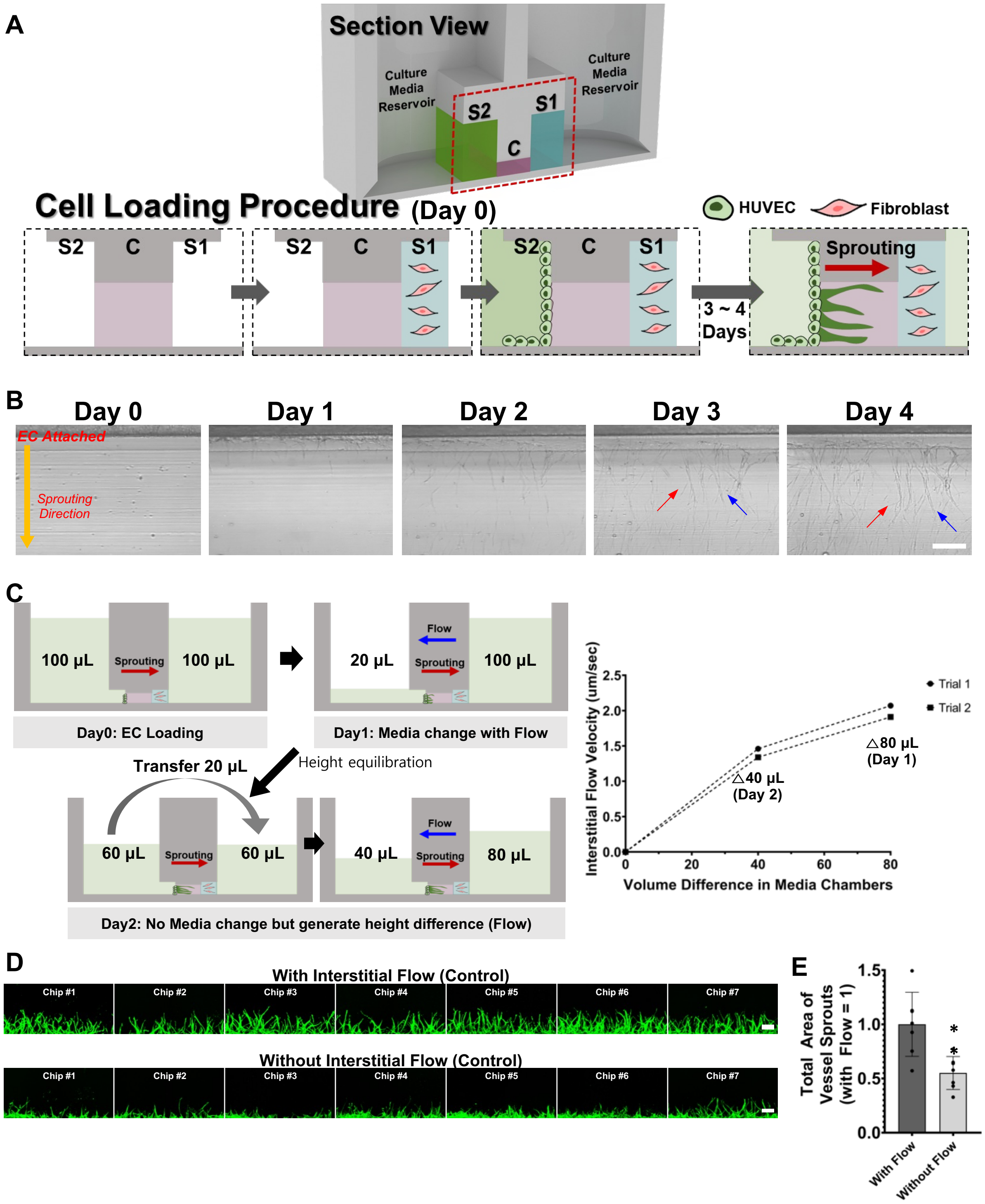

Supplementary Fig. 1. 3D angiogenic sprouting assay using microfluidic angiogenesis-on-a-chip. (A) Schematic illustration of cell loading procedure in angiogenesis-on-a-chip. In day 0, 3D fibrin hydrogel is loaded on Channel C (pink), fibroblasts mixed with 3D hydrogel are patterned on Channel S1 (blue), and HUVEC suspension is applied to form monolayer between Channels S2 and C (green). 3D angiogenic sprouts are gradually generated from day 1 and measured at day 4 for the analysis. **(B)** Phase contrast images of the angiogenic sprouts generated from the same sample taken day-by-day. The same individual sprouts at days 3 and 4 are indicated by red and blue arrows, respectively. Scale bar, 200 μm. **(C)** Schematic illustration of how the interstitial flow was generated in the microfluidic angiogenesis-on-a-chip - 20 (Channel S2) : 100 μL (Channel S1) by media change with flow at day 1;

and 40 : 80 µL by no media change, but by transferring 20 µL from Channel S2 to S1. The height difference between Channels S2 and S1 establishes hydrostatic pressure to generate the interstitial flow from Channel S1 to S2. Measured interstitial flows using FRAP (fluorescence recovery after photobleaching) technique are also shown (right). (**D**) A series of z-projected confocal images of angiogenic sprouts generated from all EC samples cultured with or without the interstitial flow. All angiogenic sprouts are stained with anti-CD31 (green). Scale bar, 200 µm. (**E**) Quantitative analysis of total area of sprouts generated from ECs cultured with or without the flow. **$p < 0.01$, unpaired t test ($n = 7$ per condition. Biological replicates). Data are mean ± S.D. Source data and exact p-values are provided in a Source Data file.

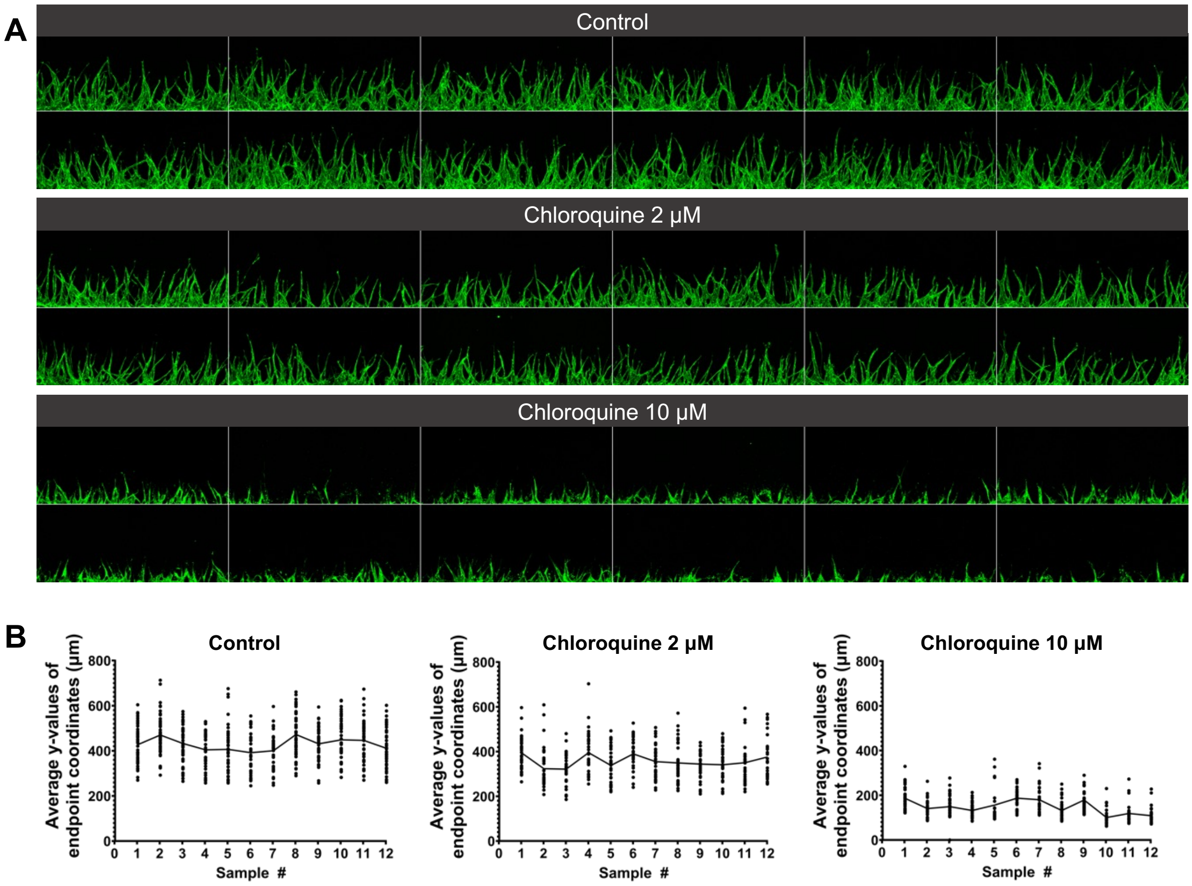

Supplementary Fig. 2. Reproducibility in angiogenic sprouting achieved by microfluidic angiogenesis-on-a-chip. (A) z-projected confocal images of angiogenic sprouts generated from all endothelial cell (EC) samples treated with each indicated concentration of chloroquine (CQ). All angiogenic sprouts are immunostained with anti-CD31(green). Scale bar, 200 µm. **(B)** Distributions of the lengths of angiogenic sprouts (y-values of sprout endpoints) generated from the individual EC samples treated with each indicated concentration of CQ. Each dot represents a single sprout, and the line represents the average values in the individual biological replicate samples. Source data are provided as a Source Data file.

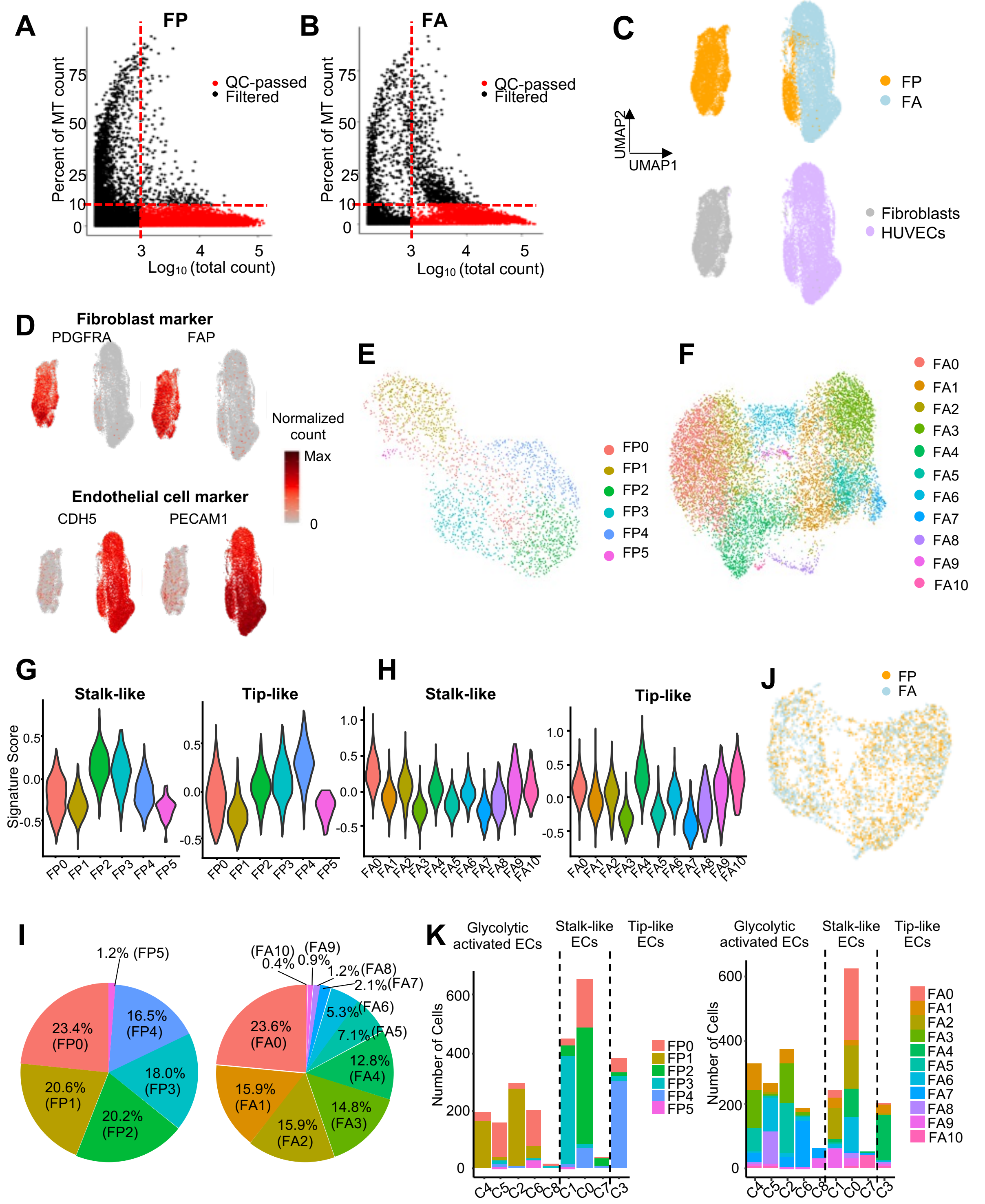

Supplementary Fig. 3. Quality control of scRNA-seq data of HUVECs in 3D angiogenesis-on-a-chip. (A, B) Scatter plots depicting the distribution of $\log_{10}$(total UMI count) and the percentage of the UMI count assigned to mitochondrial genes (MT count) in each EC under fibroblast present (FP) (**A**) and absent (FA) (**B**) conditions. Cells with $\log_{10}$(total UMI count) < the designated threshold (indicated by the red dotted line) were excluded, as were cells with the percentages of the UMI counts assigned to mitochondrial genes (MT count) > the designated threshold (indicated by the red dotted line). **(C)** UMAP plot of 22,077 ECs that met the above quality control c

riteria under FP and FA conditions. Cells are colored by their conditions (top) and cell type (bot tom). **(D)** UMAP plots showing the expression of EC (CDH5 and PECAM1) and fibroblast (PD GFRA and FAP) markers. (**E**, **F**) UMAP plot showing six clusters (FP0-5) of ECs under FP con dition (**E**) and nine clusters (FA0-8) of ECs under FA condition (**F**). Cells are colored by their c lusters. (**G**, **H**) Violin plots showing the distributions of stalk-like and tip-like signature scores of the indicated EC clusters under FP (**G**) and FA conditions (**H**). Source data and exact p-values are provided in a Source Data file. (**I**) Pie charts showing the percentages of ECs in FP0-5 unde r FP condition (left) and ECs in FA0-8 under FA condition (right). (**J**) UMAP plot showing all ECs under FP condition (blue) and the sampled ECs under FA condition (orange). (**K**) Stacked b ar graphs showing the numbers of ECs in FP and FA EC clusters belonging to each combined c luster (C0-8) under FP (left) and FA (right) conditions. Source data are provided as a Source Da ta file.

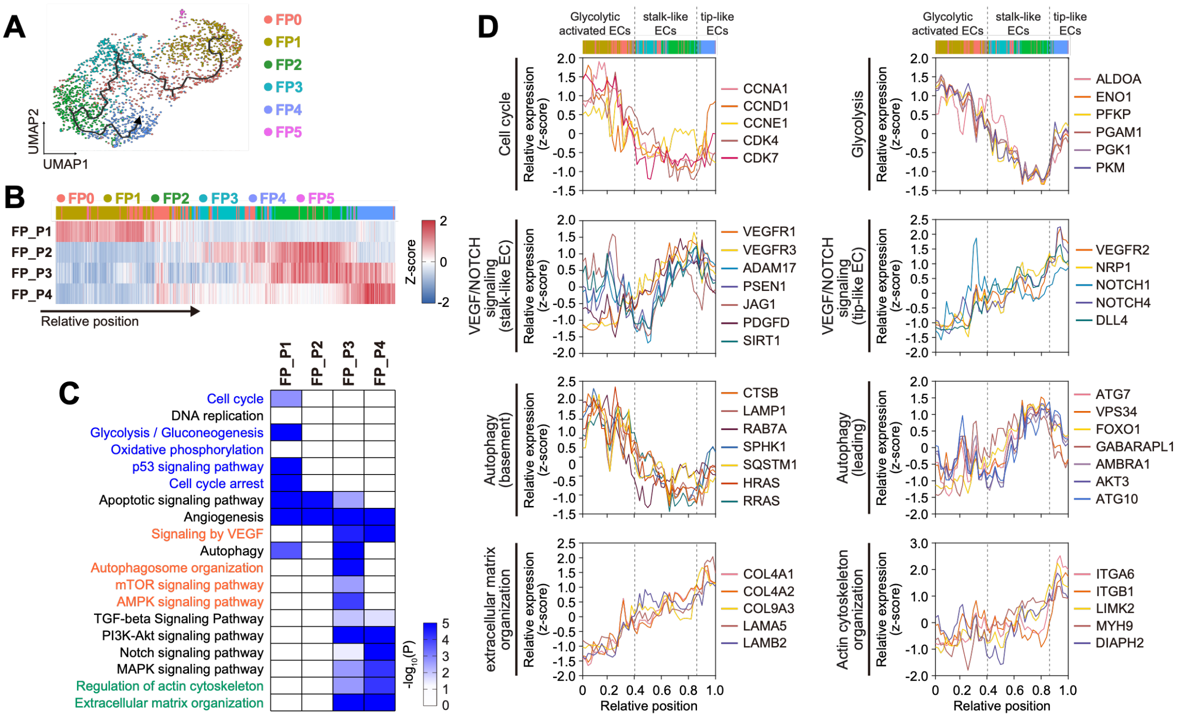

Supplementary Fig. 4. Spatially differential activations of autophagy in the basement and leading positions observed when only ECs in the presence of fibroblasts were analyzed. (A) UMAP plots showing the trajectory (up) and corresponding relative positions (down) along elongating sprouts. Linear trajectory is obtained from ECs in FA0-5. (B) Four sets of genes (FP_P1-4) showing activation in basement (FP_P1), middle (FP_P2), and leading positions (FP_P3-4) along the trajectory. The mean expression (Z-score) of genes in each set is displayed in individual ECs along the trajectory. Colored bars on the top denote cluster memberships of the individual ECs. Relative position along the trajectory increases along the elongating sprouts axis (arrow). (C) Cellular pathways enriched by the genes in FP_P1-4 for the trajectory. Enrichment significance (enrichment P-value from hypergeometric test implemented at ConsensusPathDB) for cellular pathways is displayed as $-\log_{10}(P)$. Color bar, the gradient of $-\log_{10}(P)$. (D) Relative expression profiles of the indicated representative genes involved in the indicated pathways related to cell proliferation, angiogenesis, autophagy, and tip cell migration along the trajectory. Source data are provided as a Source Data file.

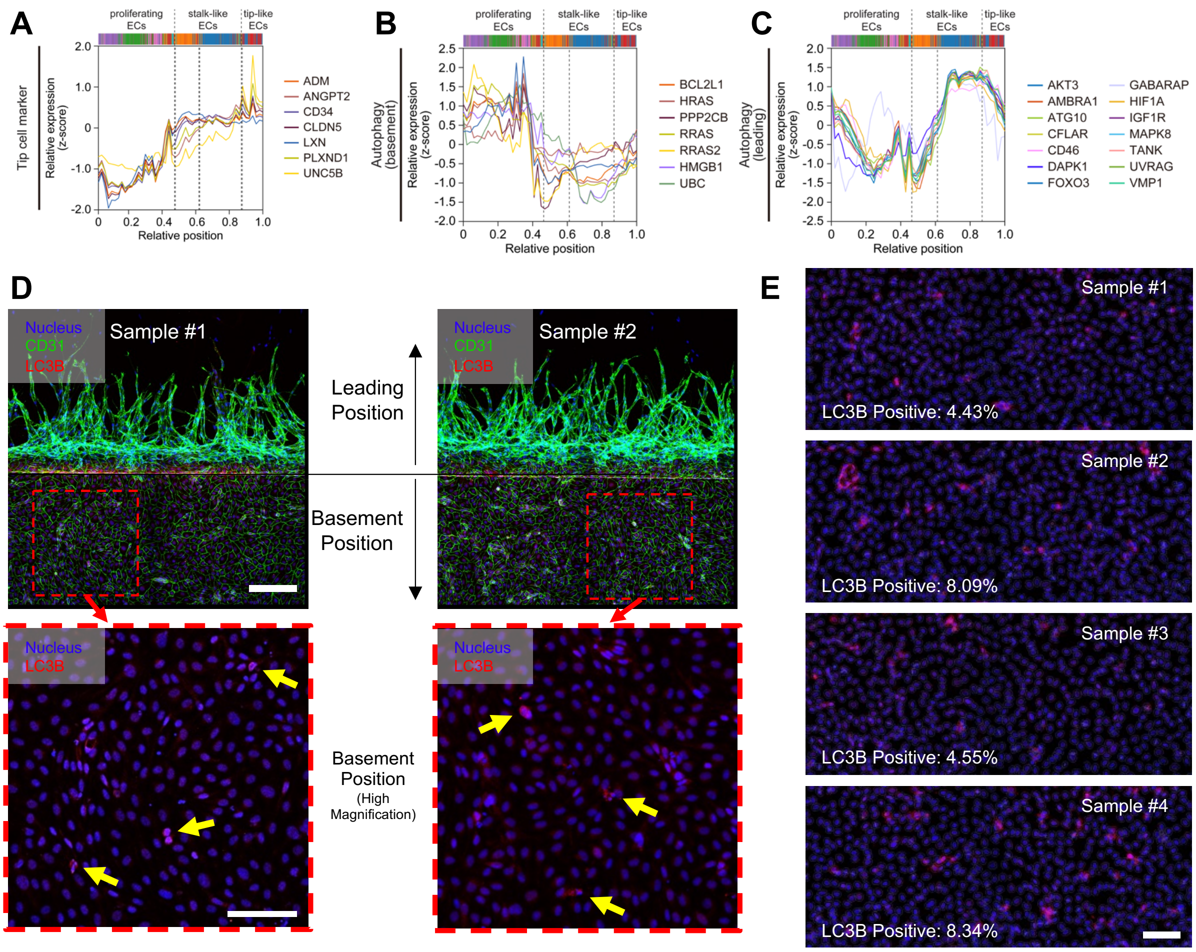

Supplementary Fig. 5. Tip cell markers in the leading positions and activation of autophagy in the basement positions. (A-C) Expression patterns of marker genes previously known to be highly upregulated in tip cells (**A**) and autophagy-related genes activated in the basement (**B**) or leading position (**C**) along the trajectory. Colored bars on the top denote cluster memberships of the individual ECs. Relative position along the elongating sprouts increases from left to right. Source data are provided as a Source Data file. **(D)** Representative z-projected confocal images of sprouts on 3D angiogenesis-on-a-chip at day 4 with immunostaining of CD31 (green), LC3B (red), and Nucleus (blue). Scale bar, 200 μm for two above images, and 100 μm for two lower magnified images of basement positions. In the basement position, ECs expressing LC3B proteins are indicated by yellow arrowheads. (E) Percentages of LC3B-expressing ECs among ECs in the basement of the representative z-projected confocal images. Scale bar, 100 μm.

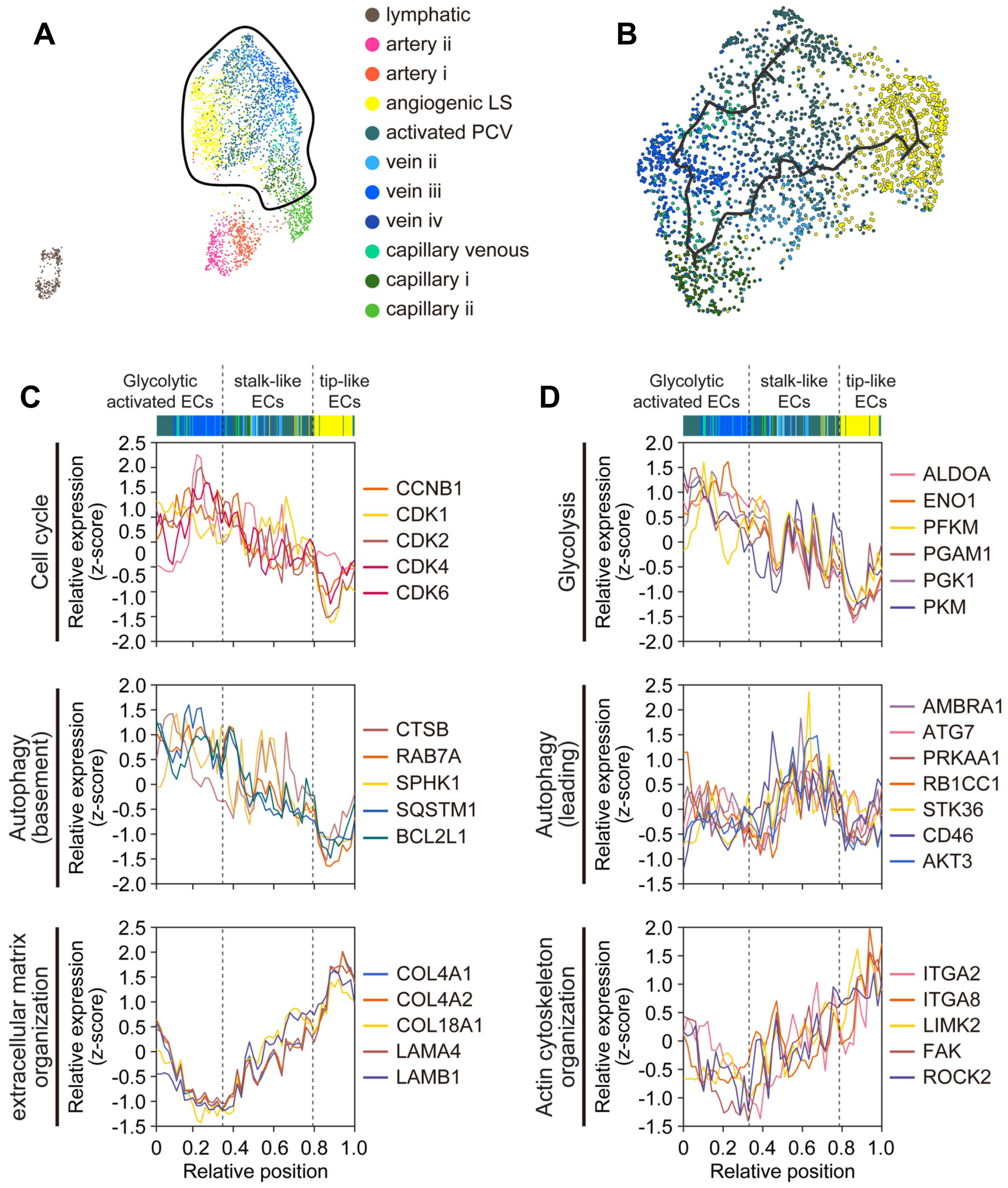

Supplementary Fig. 6. Shared characteristics of proliferating and tip/stalk-like ECs in 3D angiogenic sprouts with those in human breast cancer tissues involving angiogenesis. (**A**) UMAP plot showing ECs from tumor tissues of patients with breast cancer. Clusters used for trajectory analysis are indicated by the black line. (**B**) UMAP plot showing the trajectory (black line) estimated for the selected ECs in (**A**). (**C**, **D**) Expression profiles of the genes defining Cell cycle, Glycolysis, Autophagy (basement), Autophagy (leading), ECM, and Actin cytoskeleton in Fig. 4D and their signature score along the trajectory. Source data are provided as a Source Data file.

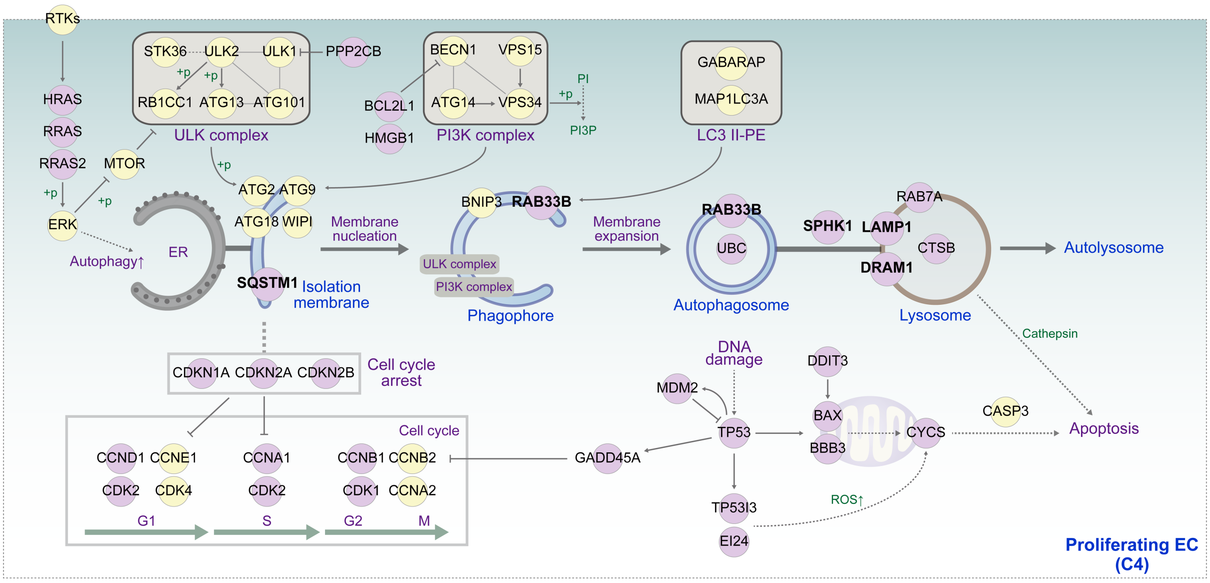

Supplementary Fig. 7. A network model describing interactions of autophagy-related molecules predominantly upregulated in proliferating ECs in the basement. Node colors represent upregulation in the basement (magenta) or no change (yellow) of the corresponding genes, respectively. Arrows and suppression symbols represent activation and inhibition in the corresponding signaling reactions, respectively. Solid and dotted lines denote direct and indirect interactions, respectively.

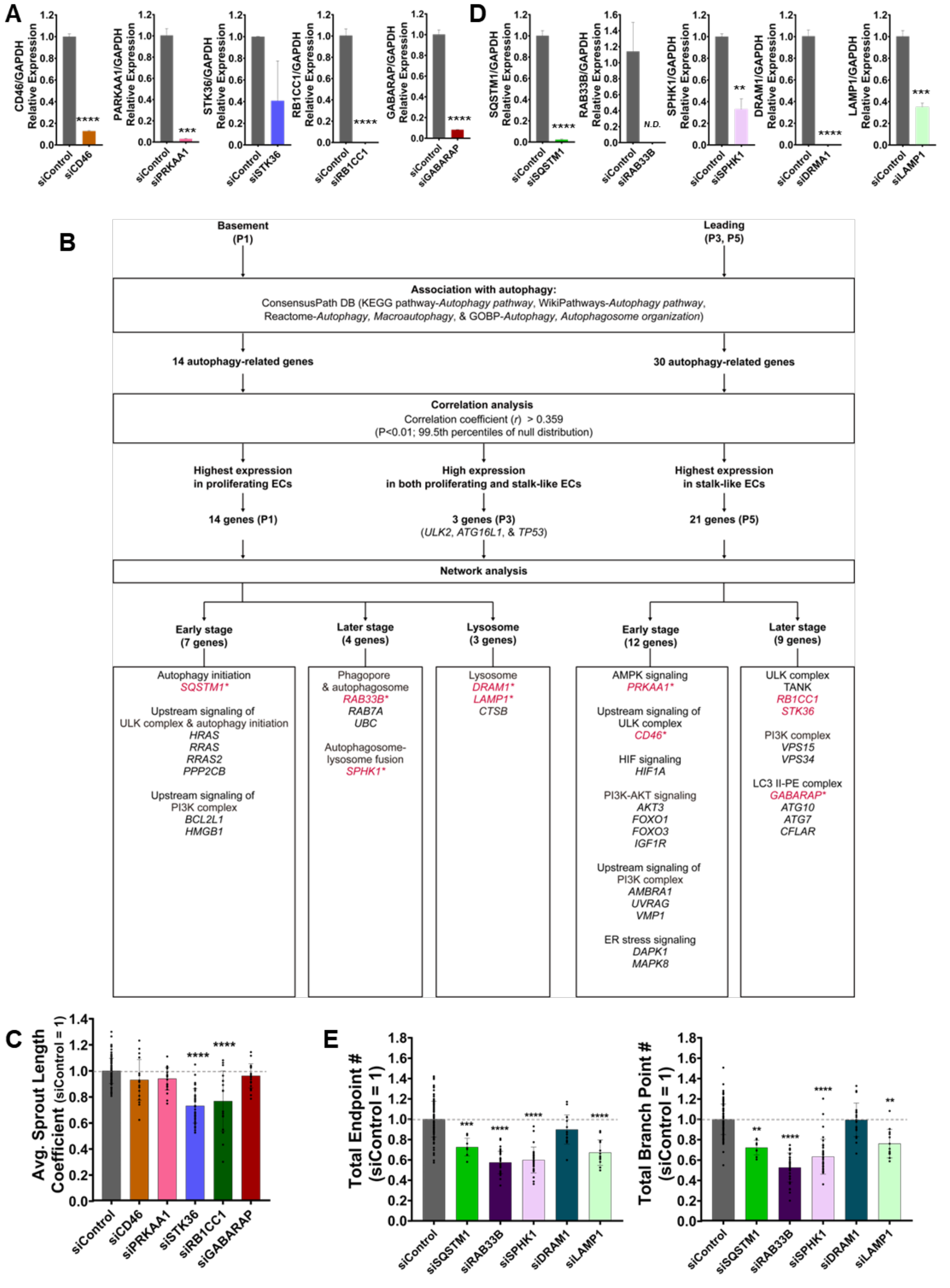

Supplementary Fig. 8. Effects of siRNA-based autophagy-related gene knockdown on angiogenic sprouting. (A, D) Decreased relative expression of mRNA in EC verified using RT-qPCR 12 h after siRNA transfection for each autophagy-related gene highly expressed in the leading **(A)** or basement **(D)** position of angiogenic sprouts. *p < 0.05, **p < 0.01, ***p < 0.001, ****p < 0.0001, n.d.: not detected, unpaired t test (n = 2 to 3 per gene). Source data and exact p-values are provided in a Source

Data file. **(B)** Schematic illustration for network-based selection of autophagy-related genes experimentally tested. We first identified autophagy-related genes from P1, 3, and 5 by which autophagy pathway was significantly enriched (Fig. 4C)- 14 genes from P1 (basement) and 30 genes from P3 or 5 (leading position). Second, among them, we then selected the following three groups of genes with similar expression patterns (Spearman's correlation $r > 0.359$; $P = 0.01$): Group 1) 14 genes with the highest expression in proliferating ECs; Group 2) 21 genes with the highest expression in stalk-like ECs; and Group 3) 3 genes with high expression in both proliferating and stalk-like ECs. The cutoff of the similarity of the expression patterns along the trajectory was determined based on the null distribution of r. The mean profiles of the expression patterns for P1 (basement) and P3 or 5 (leading position) were estimated by performing random permutation of the cell order along the trajectory. The null distribution was estimated from the correlations between the expression profiles of the genes in P1 (basement) and P3 or 5 (leading position) and randomized mean expression profiles. These steps were repeated 1,000 times. Based on the null distribution, we computed p-values for the observed r value. A network model for 14 genes in Group 1 was reconstructed to describe their regulatory relationships with upstream signaling pathways of ULK/PI3K complexes, VEGF/NOTCH signaling, and the predicted regulation of VEGFR2 proteins by autophagy in stalk-like ECs (Fig. 5B). Another model for 21 genes in Group 2 was reconstructed to describe their regulatory relationships with upstream signaling pathways of ULK/PI3K complexes, cell cycle, and apoptosis (Fig. 5C). According to these network models, the 14 genes in Group 1 (or 21 genes in Group 2) were categorized into the ones at early and late stages of autophagy or lysosome, each of which was further grouped into functional modules: For example, 12 genes in Group 2 involved in early stage of autophagy was grouped into PRKAA1 in AMPK signaling, CD46 in the upstream signaling of ULK complex, HIF1A in HIF signaling, AKT3, FOXO1/3, and IGF1R in PI3K-AKT signaling, AMBRA1, UVRAG, and VMP1 in the upstream signaling of PI3K complex, and DAPK1 and MAPK8 in ER stress signaling. Based on this functional module-based classification of the genes in the network models, we finally selected the representative gene closely associated with early and late stages of autophagy and lysosome (see genes red-highlighted in the boxes). **(C)** Average sprout lengths of angiogenic sprouts generated from ECs transfected with siRNA targeting autophagy-related genes predominantly activated in the leading position (Fig. 5B). **(E)** Total numbers of endpoint and branch point of angiogenic sprouts generated from ECs transfected with siRNA targeting autophagy-related genes predominantly activated in the basement position (Fig. 5C). $^{**}p < 0.01$, $^{***}p < 0.001$, $^{****}p < 0.0001$, One-way ANOVA with Tukey's posthoc test. $n = 99$ for siControl-transfected ECs and $n = 9$ to 39 for other siRNA-transfected ECs. Biological replicates. Data are mean ± S.D. Source data and exact p-values are provided in a Source Data file.

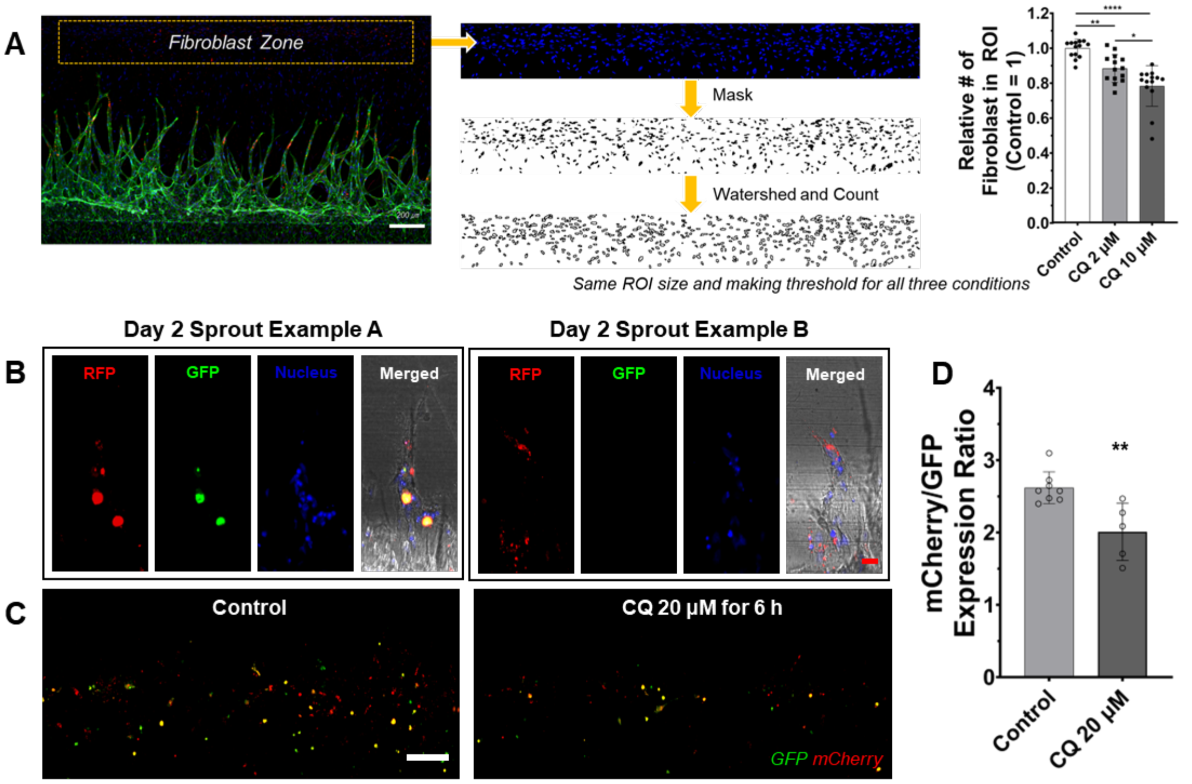

Supplementary Fig. 9. Several issues in microfluidic angiogenesis-on-a-chip. (A) Effects of CQ on the number of fibroblasts (right) present in the boundary (box in left) between Channels C and S2. *p < 0.05, **p < 0.01, ****p < 0.0001, One-way ANOVA with Tukey's posthoc test (n = 14 per condition. Biological replicates.). Scale bar, 200 µm. Data are mean ± S.D. **(B)** Representative images of the endpoint of angiogenic sprouts generated from EC transfected with LC3B-mCherry-GFP. Scale bar, 20 µm. (**C**) Representative images of the whole angiogenic sprouts generated from an EC sample treated with or without CQ 20 µM 6 h before fixing. Scale bar, 200 µm. (**D**) Quantitative analysis on the ratio of mCherry and GFP signal (mCherry/GFP) with or without CQ treatment. All samples in Supplementary fig. 8B-D are fixed at day 2 after EC loading to preserve transfection efficiency. **p < 0.01, unpaired t test (n = 8 for non-treated control and n = 5 for CQ-treated condition. Biological replicates.). Data are mean ± S.D. Source data and exact p-values are provided in a Source Data file.

Supplementary Table 1. Summary of treatment regimen of autophagy inhibitors in previous stu dies.

Inhibitor	Target Cell	Concentration	Duration	Reference
**Chloroquine**	EC	5 μM	16 h	*Lu, Haocheng, et al. Science signaling 10.464 (2017): eaah4 214.*
	EC	3 μM	24 h	*Wang, Qilong, et al. PloS one 6.2 (2011): e17234.*
	EC	25 μM	24 h	*Jeong, In-Hye, et al. Cell death & disease 11.8 (2020): 1-11.*
	U87	20 μM	24 h	*Ye, Hongxing, et al. BMC neurology 16.1 (2016): 1-8.*
	Panc	54.2 μM (IC50)	96 h	*Eng, Christina H., et al. Proceedings of the National Academy of Sciences 113.1 (2016): 182-187.*
	HCT116	37.7 μM (IC50)	96 h	*Eng, Christina H., et al. Proceedings of the National Academy of Sciences 113.1 (2016): 182-187.*
**Wortmannin**	EC	30 nM	6 h	*Alhosin, Mahmoud, et al. PLoS One 8.3 (2013): e57883.*
	EC	10 μM	24 h	*Gomes, Anderson M., et al. Journal of Cellular Physiology 23 5.6 (2020): 5256-5269.*
	EC	10 μM	1 h	*Srisook, Klaokwan, et alInflammopharmacology 28.6 (2020): 1649-1662.*
**BafilomycinA1**	EC	200 nM	16 h	*Lu, Haocheng, et al. Science signaling 10.464 (2017): eaah4 214.*
	EC	10 nM	24 h	*Wang, Qilong, et al. PloS one 6.2 (2011): e17234.*
	EC	10 nM	24 h	*Jeong, In-Hye, et al. Cell death & disease 11.8 (2020): 1-11.*
**3MA**	EC	5 mM	16 h	*Lu, Haocheng, et al. Science signaling 10.464 (2017): eaah4 214.*
	EC	2 mM	24 h	*Jeong, In-Hye, et al. Cell death & disease 11.8 (2020): 1-11.*
	EC	1 mM	24 h	*Li, Zheng, et al. Biological Trace Element Research 191.1 (20 19): 88-97.*

Supplementary Table 2. Autophagy-related genes selected for experimental testing.

			KEGG (Autophagy pathway)	Reactome (Autophagy, Macroautophagy)	WikiPathways (Autophagy pathway)	GO Biological Process (Autophagy, Autophagosome organization)	
Leading	GABARAP	11337	O	O	O	O	
	PRKAA1	5562	O	O	O	O	
	RB1CC1	9821	O	O	O	O	
	CD46	4179					Autophagy induction by the pathogen receptor CD46 (PMID: 19837375; PMID: 32582714)
	STK36	27148					One of the five ULK1 homologues (ULK1, ULK2, ULK3, ULK4 and STK36 (serine/threonine kinase 36)); ULK1 and ULK2 are believed to be involved in conventional autophagy signalling. (PMID: 29233870)
Basement	SQSTM1	8878	O	O		O	
	RAB33B	83452	O			O	
	LAMP1	3916	O		O	O	
	SPHK1	8877			O		
	DRAM1	55332				O	

Supplementary Table 3. The sequences of siRNA targeting autophagy-related genes.

siRNA	Sense (5' – 3')	Anti-Sense (5' – 3')
siControl (Negative control)	UUCUCCGAACGUGUCACGUTT	ACGUGACACGUUCGGAGAATT
siCD46	GGAUACUUCUAUAUACCUCUU	AAGAGGUAUAUAGAAGUAUCC
siPRKAA1	GGAUCCAUCAUAUAGUUCATT	UGAACUAUAUGAUGGAUCCTT
siSTK36	CUCUGCACUGGCUUCAGAUGTT	CAUCUGAAGCCAGUGCAGAGTT
siRB1CC1	CCGAGUUAAUUAGUAGACAUGAAGA	UCUUCAUGUCUACUAAUUAACUCGG
siGABARAP	CCGGGUGCCGGUGAUAGUAGA	UCUACUAUCACCGGCACCCGG
siSQSTM1	GUGACGAGGAAUUGACAAUTT	AUUGUCAAUUCCUCGUCACTT
siRAB33B	GAUAUACCACGGUUCUUGUUTT	AACAAGAACCGUGGUAUAUCTT
siSPHK1	GAGCUGCAAGGCCUUGCCCTT	GGGCAAGGCCUUGCAGCUCTT
siLAMP1	AGAAAUGCAACACGUUATT	UAACGUGUUGCAUUUCUTT
siDRAM1	CCACGAUGUAUACAAGAUATT	UAUCUUGUAUACAUCGUGGTT
siATG5	GGAUGCAAUUGAAGCUCAUTT	AUGAGCUUCAAUUGCAUCCTT
siATG7	GGAACACUGUAUAACACCATT	UGGUGUUAUACAGUGUUCCTT

Supplementary Table 4. Primer sequences for quantitative real-time PCR (qRT-PCR)

Gene (Human)	Forward Primer (5' – 3')	Reverse Primer (5' – 3')
GAPDH	CGCTGAGTACGTCGTGGAGT	AGAGGGGGCAGAGATGATG
CD46	TGGCTACCTGTCTCAGATGACG	GCATCTGATAACCAAACTCGTAAG
PRKAA1	TCAGGAAGATTGTATGCAGGCCCA	TTCATGGGATCCACCTGCAGCATA
STK36	GCGAGTAAAATTGCGTCCCA	CTCAGCTCCTTCTCTGAGCG
RB1CC1	CCTAGACGAACGCCATGACA	AGCTGGTTTGAGATCCAGGG
GABARAP	GCTCCCAAAGCTCGGATAG	TGGGTGGAATGACATTGTTG
SQSTM1	GAACTCCAGTCCCTACAGATGCC	CGGGAGATGTGGGTACAAGG
RAB33B	AGAACGAGCGGTGGAGATT	CCATGCTCTTTCTGAATCGTT
SPHK1	AGCTTCCTTGAACCATTATGCTG	AGGTCTTCATTGGTGACCTGCT
LAMP1	CTCTAATGTCTGCAGCTCAAGG	TGTACACAGCGCAGAACAGG
DRAM1	GATTGGTGGGATGTTTCGG	GAGATGATGGACTGTAGGAGC
