# Supplementary material for: Angiogenesis-on-a-chip coupled with single-cell RNA sequencing reveals spatially differential activations of autophagy along angiogenic sprouts
Source: Nat Commun. 2024 Jan 3;15:230. doi: 10.1038/s41467-023-44427-0 (PMC10764361; doi:10.1038/s41467-023-44427-0)
Supplement: Supplementary file 2 — Description of additional supplementary files [file 41467_2023_44427_MOESM2_ESM.pdf]

Description of additional supplementary files

Supplementary Data 1. List of genes included in P1-4 or FP_P1-4. **(A)** The 1,301 genes included in the P1-4. **(B)** The 512 genes included in the FP_P1-4. See the excel file entitled "Supplementary Data 1".
