# Supplementary material for: Angiogenesis-on-a-chip coupled with single-cell RNA sequencing reveals spatially differential activations of autophagy along angiogenic sprouts
Source: Nat Commun. 2024 Jan 3;15:230. doi: 10.1038/s41467-023-44427-0 (PMC10764361; doi:10.1038/s41467-023-44427-0)
Supplement: Supplementary file 4 — Reporting Summary [file 41467_2023_44427_MOESM4_ESM.pdf]

nature portfolio

Corresponding author(s): Noo Li Jeon, Sung Hee Baek, Daehee Hwang, and Jong Kyoung Kim

Last updated by author(s): Nov 23, 2023

Reporting Summary

Nature Portfolio wishes to improve the reproducibility of the work that we publish. This form provides structure for consistency and transparency in reporting. For further information on Nature Portfolio policies, see our Editorial Policies and the Editorial Policy Checklist.

Statistics

For all statistical analyses, confirm that the following items are present in the figure legend, table legend, main text, or Methods section.

n/a	Confirmed	
☐	☒	The exact sample size (*n*) for each experimental group/condition, given as a discrete number and unit of measurement
☐	☒	A statement on whether measurements were taken from distinct samples or whether the same sample was measured repeatedly
☐	☒	The statistical test(s) used AND whether they are one- or two-sided<br>*Only common tests should be described solely by name; describe more complex techniques in the Methods section.*
☐	☒	A description of all covariates tested
☐	☒	A description of any assumptions or corrections, such as tests of normality and adjustment for multiple comparisons
☐	☒	A full description of the statistical parameters including central tendency (e.g. means) or other basic estimates (e.g. regression coefficient) AND variation (e.g. standard deviation) or associated estimates of uncertainty (e.g. confidence intervals)
☐	☒	For null hypothesis testing, the test statistic (e.g. *F*, *t*, *r*) with confidence intervals, effect sizes, degrees of freedom and *P* value noted<br>*Give P values as exact values whenever suitable.*
☒	☐	For Bayesian analysis, information on the choice of priors and Markov chain Monte Carlo settings
☒	☐	For hierarchical and complex designs, identification of the appropriate level for tests and full reporting of outcomes
☒	☐	Estimates of effect sizes (e.g. Cohen's *d*, Pearson's *r*), indicating how they were calculated

Our web collection on statistics for biologists contains articles on many of the points above.

Software and code

Policy information about availability of computer code

Data collection	3D confocal imaging data were collected with Nikon Eclipse Ti2-E (Nikon).<br>Real-time quantitative PCR data were collected with StepOnePlus (Applied Biosystems).<br>Libraries for scRNA-seq were pooled and sequenced through the Hiseq2500 platform (Illumina).
Data analysis	Graphpad Prism 9 was used to generate plots for quantification data and statistical analysis (t-test and one-way ANOVA with subsequent Tukey's multiple comparisons post-test).<br>Fiji (NIH) was used to measure fluorescence signal intensity of autophagy markers and endothelial markers.<br>Python 3.7.10, OpenCV and scikit-image library were used to pre-process image and quantify the vessel parameters from projection of 3D vessel confocal images.<br>MATLAB was used for hierarchical clustering (Fig.4A; clustergram.m in R2020a; www.mathworks.com/; Euclidean distance as a dissimilarity measure and ward linkage method).<br>Cell Ranger software (v.2.0.0) and packages mentioned below were used in pre-processing and analysis of scRNA-seq data.<br>R packages: scater (v.1.22.0) , scran (v.1.22.1), Seurat (v.4.3.0), monocle3(v.1.3.1)<br>Code availability: The R script used in this study is available at https://github.com/CB-postech/scRNA-HUVECs/.

For manuscripts utilizing custom algorithms or software that are central to the research but not yet described in published literature, software must be made available to editors and reviewers. We strongly encourage code deposition in a community repository (e.g. GitHub). See the Nature Portfolio guidelines for submitting code & software for further information.

Data

Policy information about availability of data

All manuscripts must include a data availability statement. This statement should provide the following information, where applicable:

- Accession codes, unique identifiers, or web links for publicly available datasets
- A description of any restrictions on data availability
- For clinical datasets or third party data, please ensure that the statement adheres to our policy

Data availability: The Single-cell transcriptomic data generated in this study have been deposited in the Sequence Read Archive (SRA) database under accession code PRJNA931762 [https://www.ncbi.nlm.nih.gov/bioproject/791201]. The publicly available breast cancer EC data used in this study are available in the gene expression omnibus database (GEO) database under accession code GSE155109 [https://0-www-ncbi-nlm-nih-gov.brum.beds.ac.uk/geo/query/acc.cgi?acc=GSE155109]. We used publicly available breast cancer EC data, which were downloaded from the gene expression omnibus database (GEO), under accession code GSE15510941.
Other data generated in this study are provided in the Supplementary Information and Source Data file.

Human research participants

Policy information about studies involving human research participants and Sex and Gender in Research.

Reporting on sex and gender	N/A
Population characteristics	N/A
Recruitment	N/A
Ethics oversight	N/A

Note that full information on the approval of the study protocol must also be provided in the manuscript.

Field-specific reporting

Please select the one below that is the best fit for your research. If you are not sure, read the appropriate sections before making your selection.

☒ Life sciences ☐ Behavioural & social sciences ☐ Ecological, evolutionary & environmental sciences

For a reference copy of the document with all sections, see nature.com/documents/nr-reporting-summary-flat.pdf

Life sciences study design

All studies must disclose on these points even when the disclosure is negative.

Sample size	The sample sizes were minimum 6 to maximum 99 in vessel morphology analysis and 3 in RT-qPCR analysis. Precise sample number in each experimental condition is stated in the legend of each figure. Sufficient number of samples for statistical evaluation of sprout morphology was determined based on the result in Supplementary Figure 2, showing consistent distributions of both sprout length and width across different samples under the same culture condition. In addition, recently published works utilizing organ-on-a-chip have similar sample number (e.g. Dasgupta et al., Nature Communications, (2023) 14:6506).
Data exclusions	Data was not excluded from experiments unless apparent failures, such as 3D hydrogel 'patterning' failure during the 'initial' step of 3D EC sprouting assay.
Replication	All experiments, such as autophagy-related drug treatment experiment and siRNA-based gene regulated HUVEC sprouting experiment, were replicated with at least 2 chip set-ups, and all attempts at replication were successful as the tendency of the change in sprouting was consistent. To ensure the reproducibility, 28 individual samples in chip culture were collected and pooled per condition for single-cell RNA sequencing.
Randomization	Samples were allocated into experimental group at random.
Blinding	The investigators were blinded to group allocation during experiments and data collection. Same imaging setting was used for all samples for quantitative comparison analysis.

Reporting for specific materials, systems and methods

We require information from authors about some types of materials, experimental systems and methods used in many studies. Here, indicate whether each material, system or method listed is relevant to your study. If you are not sure if a list item applies to your research, read the appropriate section before selecting a response.

Materials & experimental systems

n/a	Involved in the study
☐	☒ Antibodies
☐	☒ Eukaryotic cell lines
☒	☐ Palaeontology and archaeology
☒	☐ Animals and other organisms
☒	☐ Clinical data
☒	☐ Dual use research of concern

Methods

n/a	Involved in the study
☒	☐ ChIP-seq
☒	☐ Flow cytometry
☒	☐ MRI-based neuroimaging

Antibodies

Antibodies used

Alex Flour 488- or 594-conjugated mouse anti-human cluster of differentiation 31 (anti-CD31, Biolegend Cat. 303110 and 303126, 1:200, Clone: WM59)
Rabbit anti-human LC3B antibody (Abcam,Cat. ab48394, 1:200, polyclonal)
Alexa Fluor 568 goat anti-rabbit immunoglobulin G (IgG) (Invitrogen, Cat. A-11036, 1:500, polyclonal)

Validation

All antibodies used in this work was validated by company for Immunocytochemistry (ICC/IF) application. Recommended concentration and references of reported studies are listed in the downloadable data sheet in each website.

Alex Flour 488- or 594-conjugated mouse anti-human CD31 are verified with Human, Cynomolgus, Rhesus samples.
https://www.biolegend.com/en-gb/products/alexa-fluor-488-anti-human-cd31-antibody-3259?GroupID=BLG10311
https://www.biolegend.com/en-gb/products/alexa-fluor-594-anti-human-cd31-antibody-10182

Rabbit anti-human LC3B antibody is verified with Mouse, Rat, Human samples.
https://www.abcam.com/products/primary-antibodies/lc3b-antibody-autophagosome-marker-ab48394.html

Alexa Fluor 568 goat anti-rabbit IgG are verified with rabbit samples.
https://www.thermofisher.com/antibody/product/Goat-anti-Rabbit-IgG-H-L-Highly-Cross-Adsorbed-Secondary-Antibody-Polyclonal/A-11036

Eukaryotic cell lines

Policy information about cell lines and Sex and Gender in Research

Cell line source(s)

Human umbilical vein endothelial cells (HUVEC, C2519A, Lonza, Pooled Donor with both male and female mixed). Normal human lung fibroblasts (LF, CC-2512, Lonza, single male donor)

Authentication

HUVEC and LF was authenticated by Lonza provided with Certificate of Analysis.
HUVECs passed the qualification was guaranteed for all lots of this product using Lonza's Clonetics (TM) and Poietics (TM) Media, Reagents, and Protocols: Total Population Doublings >=15.
LF was guaranteed for all lots of this product using Lonza's Clonetics(TM) and Poietics(TM) Media, Reagents, and Protocols: Total Population Doublings >=15, Factor VIII Negative, Cytokeratin 18 Negative, Cytokeratin 19 Negative.

Mycoplasma contamination

All cells test negative for mycoplasma, bacteria, yeast, and fungi, which was performed by company.

Commonly misidentified lines
(See ICLAC register)

No commonly misidentified cell lines were used in this study.
